# Uncoupling *N*-acetylaspartate from brain pathology: implications for Canavan disease gene therapy

**DOI:** 10.1007/s00401-017-1784-9

**Published:** 2017-11-07

**Authors:** Georg von Jonquieres, Ziggy H. T. Spencer, Benjamin D. Rowlands, Claudia B. Klugmann, Andre Bongers, Anne E. Harasta, Kristina E. Parley, Jennie Cederholm, Orla Teahan, Russell Pickford, Fabien Delerue, Lars M. Ittner, Dominik Fröhlich, Catriona A. McLean, Anthony S. Don, Miriam Schneider, Gary D. Housley, Caroline D. Rae, Matthias Klugmann

**Affiliations:** 1 Translational Neuroscience Facility and Department of Physiology, School of Medical Sciences, UNSW Sydney, Sydney, NSW 2052 Australia; 2 Neuroscience Research Australia, Barker St, Randwick, NSW 2031 Australia; 3 Biomedical Imaging Resources Laboratory, Mark Wainwright Analytical Centre, UNSW Sydney, Sydney, NSW 2052 Australia; 4 Bioanalytical Mass Spectrometry Facility, Mark Wainwright Analytical Centre, UNSW Sydney, Sydney, NSW 2052 Australia; 5 Transgenic Animal Unit, Mark Wainwright Analytical Centre, UNSW Sydney, Sydney, NSW 2052 Australia; 6 Dementia Research Unit, UNSW Sydney, Sydney, NSW 2052 Australia; 7 Department of Anatomical Pathology, The Alfred Hospital, Melbourne, VIC Australia; 8 Prince of Wales Clinical School, UNSW Australia, Level 2, C25 Lowy Building, Sydney, NSW 2052 Australia; 9 Institute of Psychopharmacology and Research Group Developmental Neuropsychopharmacology, Central Institute of Mental Health, Medical Faculty Mannheim, Heidelberg University, 68159 Mannheim, Germany

**Keywords:** *N*-Acetylaspartate, Canavan disease, Myelination, White matter disorder, Brain metabolism, Neurophysiology, AAV, Gene therapy

## Abstract

*N*-Acetylaspartate (NAA) is the second most abundant organic metabolite in the brain, but its physiological significance remains enigmatic. Toxic NAA accumulation appears to be the key factor for neurological decline in Canavan disease—a fatal neurometabolic disorder caused by deficiency in the NAA-degrading enzyme aspartoacylase. To date clinical outcome of gene replacement therapy for this spongiform leukodystrophy has not met expectations. To identify the target tissue and cells for maximum anticipated treatment benefit, we employed comprehensive phenotyping of novel mouse models to assess cell type-specific consequences of NAA depletion or elevation. We show that NAA-deficiency causes neurological deficits affecting unconscious defensive reactions aimed at protecting the body from external threat. This finding suggests, while NAA reduction is pivotal to treat Canavan disease, abrogating NAA synthesis should be avoided. At the other end of the spectrum, while predicting pathological severity in Canavan disease mice, increased brain NAA levels are not neurotoxic per se. In fact, in transgenic mice overexpressing the NAA synthesising enzyme *Nat8l* in neurons, supra-physiological NAA levels were uncoupled from neurological deficits. In contrast, elimination of aspartoacylase expression exclusively in oligodendrocytes elicited Canavan disease like pathology. Although conditional aspartoacylase deletion in oligodendrocytes abolished expression in the entire CNS, the remaining aspartoacylase in peripheral organs was sufficient to lower NAA levels, delay disease onset and ameliorate histopathology. However, comparable endpoints of the conditional and complete aspartoacylase knockout indicate that optimal Canavan disease gene replacement therapies should restore aspartoacylase expression in oligodendrocytes. On the basis of these findings we executed an *ASPA* gene replacement therapy targeting oligodendrocytes in Canavan disease mice resulting in reversal of pre-existing CNS pathology and lasting neurological benefits. This finding signifies the first successful post-symptomatic treatment of a white matter disorder using an adeno-associated virus vector tailored towards oligodendroglial-restricted transgene expression.

**Electronic supplementary material:**

The online version of this article (10.1007/s00401-017-1784-9) contains supplementary material, which is available to authorized users.

## Introduction

Despite the ill-defined physiological function of *N*-acetylaspartate (NAA), this metabolite is widely used as a biomarker of neuronal integrity in magnetic resonance spectroscopy [56]. In fact, with CNS concentrations in the millimolar range, NAA is among the most abundant organic metabolites in the mammalian brain and displays a remarkably segmented metabolism. NAA is produced by N-acetyltransferase 8-like (NAT8L) from acetyl-CoA and aspartate in neurons, and hydrolysed into aspartate and acetate by aspartoacylase (ASPA) in oligodendrocytes [60].

Clinical evidence suggests that alterations in NAA metabolism are not well tolerated. The loss of NAT8L activity abolishes NAA production and has been reported for the neurodevelopmental disorder Hypoacetylaspartia, presenting with seizures, ataxia and microcephaly [52]. Conversely, lack of NAA catabolism caused by missense mutations in the *ASPA* gene results in the leukodystrophy Canavan disease (CD) characterized by a build-up of NAA in brain, blood and urine. CD is a fatal neurodegenerative disorder, where patients fail to reach developmental milestones, and presents with macrocephaly, seizures, widespread CNS vacuolization and hypomyelination [38, 49].

Due to its devastating nature and lack of therapeutic options *ASPA* gene replacement therapy has been attempted as an experimental treatment for CD patients [48]. The experimental clinical treatment using first generation viral vectors was safe but therapeutic outcomes were marginal. Novel gene therapy options matching requirements for treatment of CD are now becoming available and recombinant adeno-associated virus (AAV) vectors with cell type-specific tropism or ability to cross the blood–brain barrier have been employed in *ASPA* gene replacement studies [67]. Direct pre-emptive *ASPA* gene delivery to the neonatal mouse brain utilizing an oligodendrotropic AAV vector prevents CD up to three months of age [25]. Moreover, creating an ectopic NAA sink in neurons or astrocytes using cell type-specific AAV-*ASPA* vectors was reported to be beneficial in a CD mouse model [2, 31]. These studies indicate that neurological improvements can be achieved by expressing ASPA in any of the major CNS cell types, at least in mouse, a species that is comparably short-lived.

Identifying the role of ASPA in select cell types and tissues will provide a better understanding of the CD pathophysiology and inform on the ideal target tissues for gene therapy. In the CNS abundant oligodendroglial *Aspa* expression is undisputed, yet additional expression in neurons and microglia has been reported in rats [10, 50, 55]. *Aspa* is abundantly expressed in peripheral organs [54], and a recent report revealed immune impairments in CD mice [3] indicating that a better understanding of the contribution of extra-cerebral ASPA is required to identify the primary target tissue for therapy. Preclinical gene therapy studies, as well as insights from *Nat8l/Aspa* double-knockout mice, provide evidence that the aetiology of CD is caused by excess NAA resulting in toxicity rather than an undersupply with NAA-derived acetate causing hypomyelination [2, 32, 51, 69]. These data suggest that targeting *NAT8L* gene expression to reduce NAA production might present additional therapeutic efficacy for CD.

Here, we used comprehensive phenotyping of novel engineered mouse mutants enabling dissection of the consequences associated with elevated NAA or disrupted ASPA function. These studies showed that in the absence of ASPA, NAA levels correlated with severity of the CD-like pathology. While this germ line gene therapy approach prevented spongiform vacuolisation, complete depletion of NAA elicited abnormal neuronal recruitment and severe startle deficits. At the other end of the spectrum, utilizing mice that overexpress *Nat8l* in neurons, we demonstrate that increased NAA levels are not neurotoxic per se. Moreover, oligodendrocyte-specific *Aspa* depletion in conditional mutants (*Aspa* ^*∆OL*^) resulted in endpoints matching those observed in whole body *Aspa* knockout mice. Despite complete absence of ASPA in the CNS, disease onset was delayed and histopathology was less prominent in *Aspa* ^*∆OL*^ animals. This finding has direct therapeutic relevance, because it demonstrates that *Aspa* expression in peripheral organs lowers central NAA levels thereby protracting disease progression, yet without precluding final stage pathology. Our data also underpin that optimal treatment of CD requires therapeutic *ASPA* expression in oligodendrocytes, potentially combined with attenuated NAA production.

Finally, we translated our findings preclinically by employing intracranial AAV-*ASPA* gene replacement therapy targeting oligodendrocytes in post-symptomatic CD mice. This treatment yielded near complete regression of CNS pathology and long-term neurological and neurometabolic benefits. Taken together, this study delivers fundamental new insights in the pathomechanisms underlying CD, and represents the first successful post-symptomatic treatment of a myelin disorder based on an AAV platform engineered for oligodendrocyte-specific transgene expression.

## Materials and methods

For additional experimental details please see Supplemental Experimental Procedures.

### Animals

ASPA-deficient lacZ-knock-in *Aspa* ^*lacZ/lacZ*^ mice (AKO) where used to generate *Aspa* ^*flox/flox*^ mice via Flp-mediated recombination and deletion of the ß-geo cassette as described by [63]. *Aspa* ^*lacZ*^ and *Aspa* ^*flox*^ alleles were detected using the genotyping protocol described [54]. Crossing with *Cnp* ^*Cre/*+^ mice [45] disrupted the *Aspa* ^*flox/flox*^ allele within cells of the oligodendrocyte lineage (*Aspa* ^*∆OL*^). The KO-3582 mutant mouse strain with a targeted deletion of the *Nat8l* gene locus *Nat8l* ^*tm1(KOMP)Vlcg*^ (NKO) was obtained from the Knockout Mouse Project Repository at UC Davis. *Aspa* ^*lacZ/lacZ*^; *Nat8l* ^−*/*−^ double-knockout mutants were obtained by appropriate crosses between *Aspa* ^*lacZ/*+^ and *Nat8l* ^−*/*+^ mice. To overexpress *Nat8l* in CNS neurons in transgenic mice (ThyNAT), the flag epitope-tagged murine *Nat8l* cDNA [7] was cloned into the Thy-1.2 minigene cassette [12]. The resulting pThy-Flag-*Nat8l* was linearized and microinjected into C57Bl/6 × B6D2F1 zygotes as described [36]. Offspring used in this study were backcrossed into C57Bl/6 for at least three generations.

### AAV vector production and stereotaxic delivery

A cDNA encoding human ASPA was subcloned into an AAV2 plasmid between the 1.3 kb mouse myelin basic protein (*Mbp*) promoter and the woodchuck posttranscriptional regulatory element (WPRE) followed by a bovine growth hormone poly(A). Packaging, purification and titering of AAV vectors (serotype cy5), was performed as described previously [19, 78]. P30 mice were bilaterally injected with 1 μl containing 2 × 10^9^ vg into each, the striatum (+0.7 mm AP, ± 1.2 mm ML, − 2.6 mm), thalamus (− 1.5 mm AP, ± 1.0 mm ML, − 2.9 mm DV) and cerebellum (− 5.0 mm AP, ± 1.2 mm ML, − 3.1 mm).

### Magnetic resonance imaging, ^1^H-MR spectroscopy and body composition

Mice were anaesthetised using 1.5–2% isoflurane delivered in an 80% O_2_ and 20% air mixture. Respiration rate was monitored and body temperature maintained at 37 °C throughout the experiment. In vivo MR imaging procedure was performed under general anaesthesia on a 9.4 T Bruker BioSpec Avance III 94/20 magnetic resonance microimaging system (Bruker) equipped with a cryogenic very low temperature, closed cycle cooled RF-coil. Images were acquired with an optimized isotropic 3D T2w Rapid Acquisition with Relaxation Enhancement protocol (TurboRARE) using 8 echoes per echo-train as described previously [39] Whole brain and ventricle structures were segmented using thresholding and delineation methods provided by the 3D slicer package [22]. ^1^H-MR spectroscopy was performed in 6-months-old male mice. Spectra from a 2 × 2 × 3 mm^3^ voxel in the thalamus were acquired at an echo time of 10 ms using a PRESS single voxel sequence as described in [54]. Body composition measurements were performed in conscious animals in accordance to the manufacturers instruction using the EchoMRI-900^TM^ with A100 mouse antenna insert (Echo Medical Systems).

### Metabolite and myelin lipid profiling

Animals were euthanized at six or nine months, relevant brain regions dissected quickly. Snap frozen brain tissue was pulverized, solvent extracted [46] and the lyophilized and reconstituted aqueous phase assessed using ^1^H Nuclear magnetic resonance (NMR) spectroscopy on a Bruker AVANCE III HD 600 spectrometer fitted with a cryoprobe (TCI) and refrigerated sample changer. ^1^H spectra were acquired, both with and without decoupling ^13^C using bilev composite pulse decoupling, across an effective bandwidth of 48,000 Hz during the acquisition time, on a 30 s duty cycle. Total metabolite pool sizes of lactate, glutamate, GABA, aspartate, glutamine, alanine, NAA, creatine, myo-inositol, taurine, succinate, fumarate, acetate and NAD^+^ were determined using TOPSPIN (v3.1) from the ^1^H{^13^C-decoupled} spectra as described previously [61]. Quantification of NAAG levels was performed from the aqueous fraction of the above extraction and myelin lipids from the lipophilic phase using LC–MS/MS as described [35].

### Histology, immunohistochemistry, western blot and qRT-PCR

Standard Hematoxilin & Eosin, Cresyl Violet and Luxol Fast Blue histochemical stainings were performed on paraffin sections and digitized using Mirax (Carl Zeiss) or Aperio (Leica) slide scanners as described previously [79]. Immunofluorescent detection was performed on free-floating sections as described [80] using the following antibodies: rabbit anti-ASPA serum (in house), mouse anti-NeuN (Merck), rat anti-MBP (Abcam), mouse anti-GFAP (Cell Signaling), chicken anti-β-Gal (Abcam), mouse anti-Flag (Cell Signaling) and rabbit anti-Neurofilament 200 (Sigma) followed by the appropriate Alexa-488/594 conjugated secondary antibodies (Thermo Fisher). Immunoperoxidase detection was performed on free-floating sections as described in [15] using mouse anti-APC (Merck) and rabbit anti-NeuN (Cell Signaling), the appropriate biotinolated secondary antibody (Dianova) and a Vectastain Elite ABC kit (Vector Labs). Immunoblotting was performed as described previously [78] with the following antibodies: rabbit anti-ASPA serum, mouse anti-GAPDH (Sigma), rat anti-MBP (Abcam), rat anti-PLP aa3 and rat anti-NG2 (gift of J. Trotter) followed by the appropriate HRP-conjugated secondary antibodies (Dianova). qRT-PCR for *Aspa, Nat8l*, NAAG synthetase-I and II (*Rimklb* and *Rimkla*) and hypoxanthine phosphoribosyltransferase (*Hprt*) was performed as described [26].

### Behavioural testing and brainstem recordings

Ten-week-old mutants and age- and sex-matched wildtype littermates were used for this study. The rotarod, dowel and wire suspension tests were performed as described [33, 54]. Startle testing was conducted in a startle chamber (SR-LAB; San Diego Instruments, San Diego, USA) as described previously [65]. Pre-pulse inhibition (PPI) was calculated as the per cent decrease of the ASR magnitude in trials when the startle stimulus was preceded by a prepulse [100 × (mean ASR amplitude on pulse alone trials − mean ASR amplitude on prepulse-pulse trials)/mean ASR amplitude on pulse alone trials]. Auditory function was assessed by determining auditory brainstem response (ABR) in a sound attenuating chamber as described previously [79]. Flash visual evoked potentials (fVEP) were recorded as described [70].

### Statistics

Graphs and statistical analyses were done with GraphPad Prism 6 software (La Jolla, CA). Student’s *t* test, One-way or two-way ANOVA followed by Holm-Sidak post hoc test was used for statistical analysis as appropriate. Values are presented as the mean ± SEM and *p* < 0.05 was considered as statistically significant. Multivariate analysis was performed using Simca-P + v11 (Umetrics) as described in [83].

## Results

### Neurochemical consequences associated with altered NAA metabolism

**Fig. 1:**
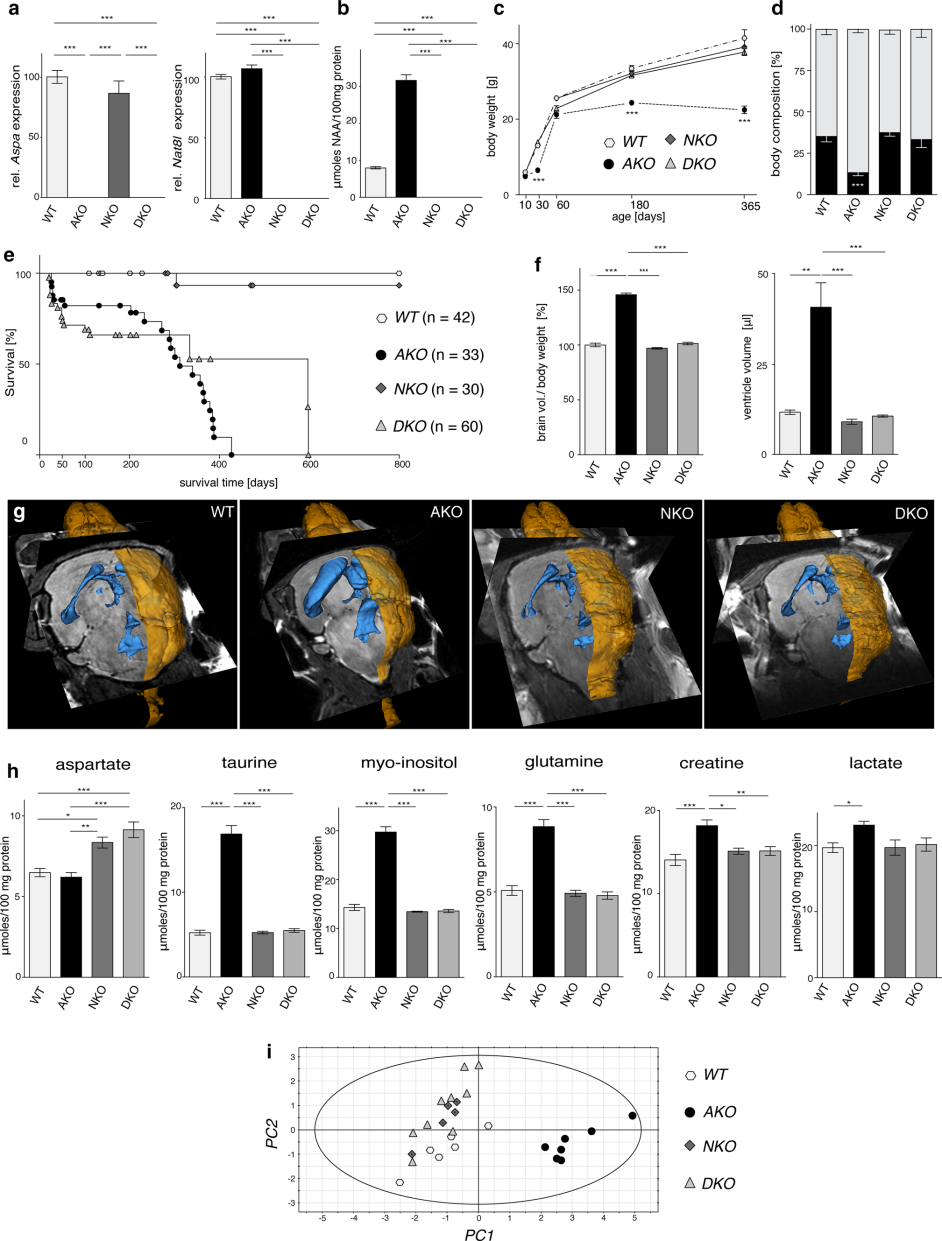
Pathological consequences of manipulation of NAA metabolism. **a** qRT-PCR analysis of *Aspa* and *Nat8l* mRNA expression in CNS tissue from DKO and the parental single gene mutants (*n* = 3; expression relative to *Hprt)*. **b** ^1^H NMR spectroscopy-based quantification of NAA levels in the brainstem of six-month-old WT, AKO, NKO and DKO mice (*n* = 6). **c** Body weight development of male WT, AKO, NKO, DKO animals (*n* = 5–14). **d** Body composition was determined in nine-month-old male mice using Echo-MRI. Both lean mass (grey) and fat mass (black) was comparable in WT, NKO and DKO mice but fat mass was significantly reduced in AKO (*n* = 3). **e** Mean life expectancy of both the AKO (312 days) and DKO (597 days) animals was diminished compared to WT and NKO mice (> 800 days). Note that the number of sudden deaths in AKO and DKO mice was particularly high during the first month of life. **f** Normalized brain volume obtained from reconstruction of T2-weighted MRIs was increased in AKO but unchanged in NKO and DKO. **g** Representative 3D reconstruction of T2-weighted MRIs from male WT, AKO, NKO and DKO brains. Segmentation of images was performed in 3D Slicer outlining brain surface (yellow) and ventricles (blue). **h** ^1^H NMR spectroscopy-based quantification of metabolite pools in the brain stem from six-month-old WT, AKO, NKO and DKO mice (*n* = 6). An increase of taurine, inositol, glutamine, creatine and lactate was found in AKO. NKO and DKO showed normal pool sizes except aspartate that was elevated in both lines. **i** Principal-component analysis of the ^1^H NMR metabolite profile in WT, AKO, NKO and DKO brainstems was performed on the significantly regulated metabolites excluding NAA. The model accounted for 98% of variance and cross-validated well with a Q2 of 90%. Data represent mean ± SEM. *p < 0.05; **p < 0.01; ***p < 0.001; One-way ANOVA with Holm-Sidak post hoc test

In order to study the effects of NAA toxicity in our *Aspa*-deficient lacZ-knock-in mouse line (AKO) [54], we ablated both alleles of the NAA producing *Nat8l* gene in the AKO line to produce double-knockout (DKO) mice. *Nat8l* deficient mice (NKO) with functional *Aspa* expression were utilized to investigate the consequences of NAA depletion and to model hypoacetylaspartia. qRT-PCR analysis of whole brain RNA confirmed absence of *Nat8l* or *Aspa* mRNA expression in DKO and the respective single gene mutants (Fig. 1a). NAA was increased in AKO (4.8 ± 0.2-fold) and not detected in NKO and DKO mice (Fig. 1b). NKO and DKO mice were born at Mendelian ratios and generally appeared and developed normally with body weight comparable to wildtype (WT) controls. AKO mice were much lighter at one month of age, caught up by two month, but plateaued from that time onward (Fig. 1c). As NAA has been attributed to lipid turnover and energy expenditure [57], we investigated the effects of genetic NAA imbalances on whole body composition (Fig. 1d). While NAA null NKO mice showed normal muscle and adipose tissue mass, we observed a strong reduction in both lean and particularly fat mass in high NAA AKO. This deficit was restored in DKO mice. The survival rate of AKO and DKO mice dropped steeply during the first 6 weeks of life (Fig. 1e). Beyond this time the maximum longevity of DKO was extended compared to AKO, but despite the lack of an obvious pathology survival did not exceed 600 days. We observed normal longevity of NKO mice.

Assessment of high resolution in vivo T_2_-weighted MRI scans saw no differences in the brain of nine-month-old DKO mice compared to NKO or WT animals. In contrast, AKO mice presented with an increase in both whole brain and ventricle volume (Fig. 1f, g). In line with this finding and concomitant to the observed NAA elevation, we detected a notable increase of organic osmolytes including myo-inositol, taurine and glutamine as well as the important energy metabolites lactate and creatine in the brainstem of AKO mice using ^1^H-NMR spectroscopy (Fig. 1h). Multivariate analysis including all pools, except NAA revealed a robust segregation of AKO from the other three groups (Fig. 1i). The NKO and DKO cohort clustered differently from WT, which could be attributed largely to a distinct elevation in the aspartate pools in these strains. Substantially decreased numbers of neurons and oligodendrocytes were detected in the thalamus of AKO, a region that shows a high degree of vacuolization (Fig. S1). Cell counts of NKO and DKO compared to controls.

Our initial findings of a near normal phenotype of our DKO mutants suggested that the NAA deletion is beneficial in CD. We then generated AKO mice with only one Nat8l allele and confirmed that the CD-associated pathology can be modulated from severe to mild by varying NAA levels in vivo (Fig. S2).

### NAA-deficiency results in neurophysiological abnormalities

**Fig. 2:**
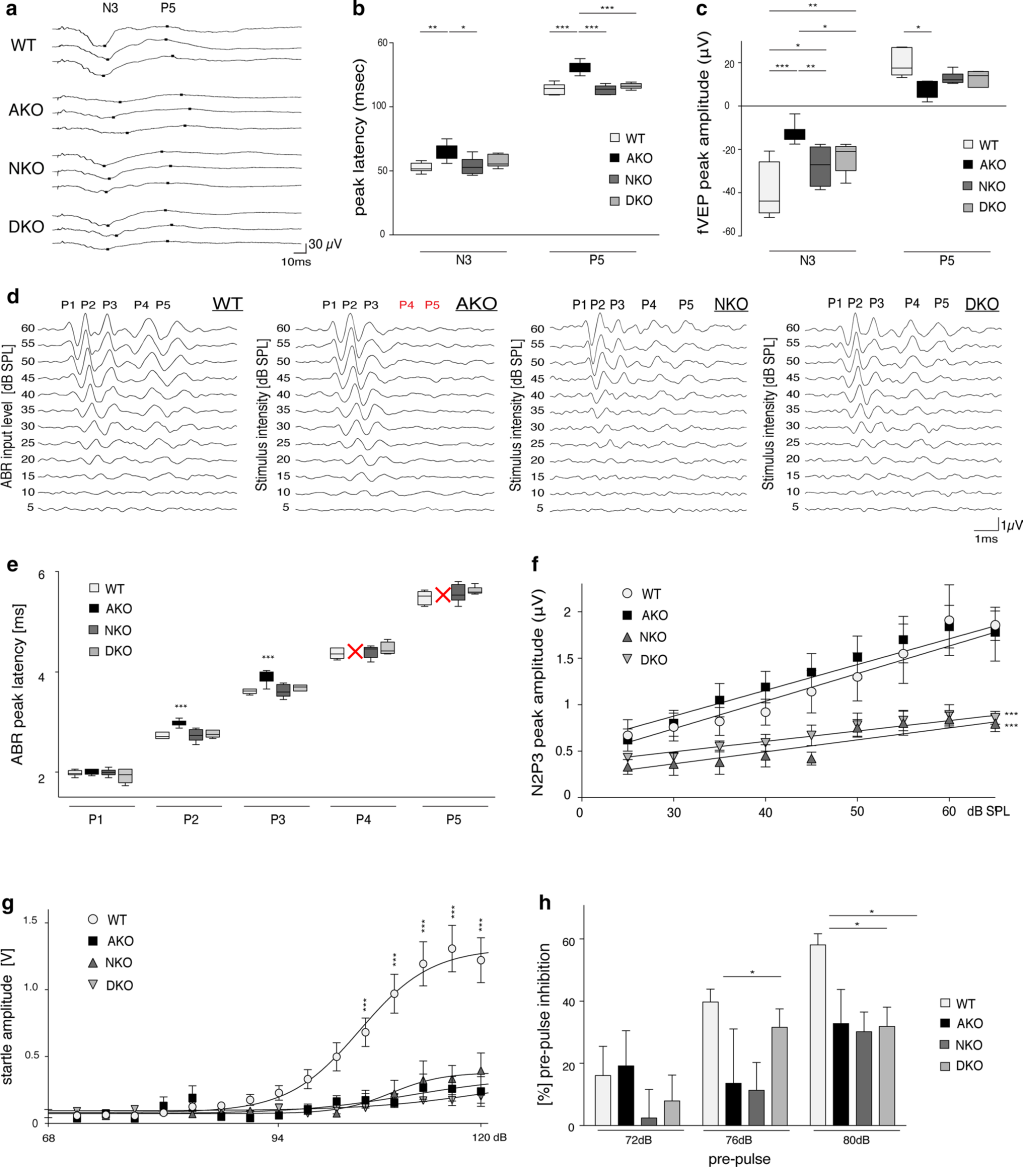
Neurophysiological consequences of Nat8l-deficiency. **a** Representative fVEP traces highlighting the location of two principal peaks N3 and P5 in WT, AKO, NKO and DKO mice. **b** Quantification of peak latencies assessed in **a** showed an increase in AKO but no change in NKO and DKO (*n* = 6). **c** Quantification of fVEP amplitudes assessed in **a** showed a decrease in all groups but did not reach statistical significance for NKO and DKO at P5. **d** Representative traces of the auditory brainstem response (ABR) in 10-week-old mice (*n* ≥ 6) following a 16 kHz tonepip. Note the absence of waves P4 and P5 in AKO mice, which was restored in DKO. **e** Quantification of wave peak latencies assessed in **d** showed an increase for P2 and P3 in AKO. P4 and P5 were completely abolished in AKO. Latencies were unchanged in NKO and DKO. **f** Input–output growth functions of N2 - P3 from the ABR data shown in **d**. Linear regression analyses are depicted for stimulus levels between 25 and 60 dB SPL. **g** Acoustic startle response of 10-week-old mice revealed severe deficits in startle amplitude of AKO (*n* = 5), NKO (*n* = 12) and DKO animals (*n* = 17) compared to WT littermates (*n* = 28). **h** Attenuated pre-pulse inhibition (PPI) in NKO and DKO mice compared with controls. Data represent mean ± SEM. *p < 0.05; **p < 0.01; ***p < 0.001; One-way ANOVA with Holm-Sidak post hoc test. *SPL* sound pressure level, *fVEP* flash visual evoked potentials

First line phenotyping of our DKO mice suggested that disease is prevented if NAA is abolished. However, for a more comprehensive assessment of the translational potential of these results we sought to investigate the neurophysiological consequences of NAA depletion. We employed flash visual evoked potentials (fVEPs), a technique that has been instrumental in identifying dysfunction of the optic system in hypomyelinating conditions [47]. Trace analysis focused on the N3 negative wave (at 52.2 ± 1.4 ms) and the P5 peak (at 98.2 ± 1.4 ms) (Fig. 2a). Indeed, peak latencies were significantly increased in AKO but unchanged from WT in NKO and DKO (Fig. 2b). The restored fVEP latencies in DKO mice confirmed normal myelination of optic nerve and optic tract. These results support our histological and biochemical data on preserved myelination in this line (Fig. S2). Importantly, quantification of the N3 peak amplitude not only revealed a decrease in AKO but also for NKO and DKO, compared with WT controls (Fig. 2c). Reduction of the P5 amplitude in NKO and DKO also approached statistical significance. A decrease in fVEP amplitudes can be attributed to a reduction in the number or sensitivity of neurons in the retinocortical pathway and is an indicator of visual impairments.

We then investigated sound-evoked auditory brainstem responses (ABR) that are known to have a short latency of 5 ms in controls (Fig. 2d). In AKO mice hypomyelination along the central auditory pathway has been reported to cause substantial delays in mid- to late-ABR waves [79]. Analysis of the ABR waveform stimulated by a 16 kHz tonepip in AKO confirmed increased latency of waveform peak 2 and 3 and absence of peak 4 and 5 compared to WT. NKO and DKO showed no alterations in peak latencies compared with controls, again reflecting normal myelination (Fig. 2e). However, the dynamic range (growth function) of the ABR was significantly decreased in DKO and NKO groups, suggesting that NAT8L-deficiency causes impaired neural recruitment in the auditory brainstem pathway. No significant differences were observed in the AKO group dynamic range compared with WT controls (Fig. 2f).

To further study sensorimotor information processing along the ponto-medullary brainstem axis towards motor neurons, we examined the acoustic startle response (ASR). NKO, AKO and DKO mice alike presented with a severely diminished startle reflex, compared to WT controls (Fig. 2g). While in AKO ASR reduction could be attributed to increased latency in auditory processing (Fig. 2e) and impaired locomotor performance (Fig. S2g), NKO and DKO performed normally in both aspects. In contrast to other facets of the CD pathology that were restored in DKO, the ASR was greatly diminished in DKO and NKO mice. Moreover, the number and appearance of giant neurons of the caudal pontine reticular nucleus, a critical relay station in the ASR pathway, was unchanged (Fig. S3). As anxiety is known to modulate the ASR amplitude [41], we employed the elevated-plus maze test, but did not observe differences between NKO, DKO and WT (not shown). We then assessed sensorimotor gating in the pre-pulse inhibition (PPI) paradigm and observed attenuated PPI amplitudes in NKO and DKO indicating cognitive or psychiatric impairment frequently observed in neurodegenerative disorders [75] (Fig. 2h).

### Transgenic mice with increased brain NAA levels are neurologically normal

**Fig. 3:**
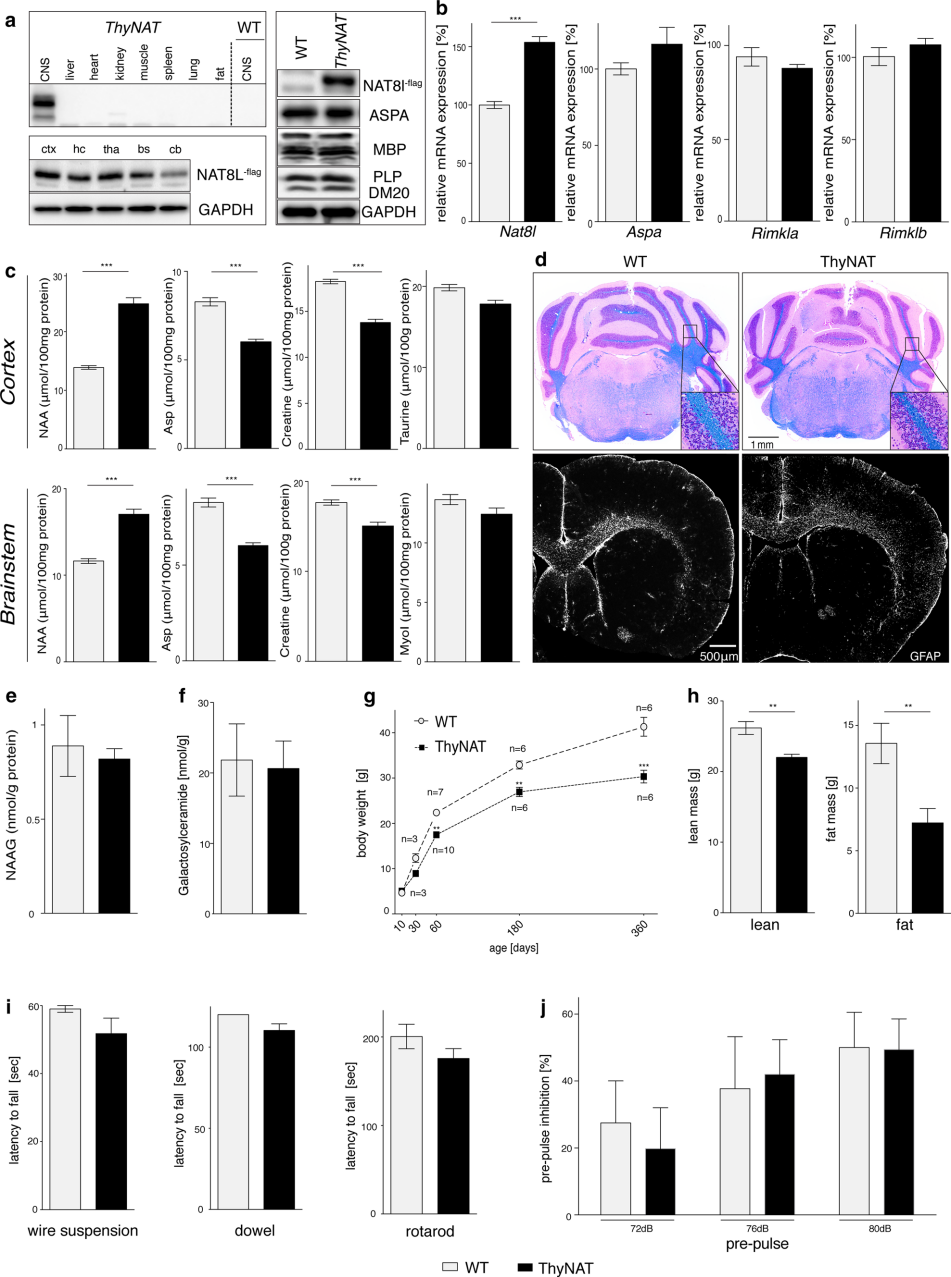
Neuronal NAT8L overexpression results in elevated NAA levels but does not cause brain damage. **a** Representative immunoblots confirming that NAT8L-flag transgene expression is restricted to the CNS. In ThyNAT mice NAT8L-flag expression is abundant in cortex (ctx), hippocampus (hc), thalamus (tha), brainstem (bs) and cerebellum (cb). The levels of ASPA, MBP or PLP appear unchanged in ThyNAT whole brain extracts (*n* = 3). **b** qRT-PCR confirms the increase of *Nat8l* mRNA expression in ThyNAT animals compared to WT. Expression of key genes associated with NAA metabolism was not significantly affected. **c** ^1^H NMR analysis confirmed increased NAA concentration in ThyNAT cortex and brainstem alongside a reduction in aspartate and creatine levels. Organic osmolytes, including taurine and myo-inositiol, remained unchanged. **d** Representative H&E/LFB histology (top) shows absence of vacuolization and normal appearance of myelin in ThyNAT mice. GFAP immunohistochemistry (bottom) revealed absence of astrogliosis in transgenic mice. **e** NAAG concentration in ThyNAT brain stem was comparable to controls. **f** Cortical concentrations of the major myelin lipid galactosylceramide were similar in transgenic and controls. **g** Body weight development of male ThyNAT animals. Starting from two months of age, transgenics remained significantly lighter than WT littermates (*n* = 3–10). **h** Body composition was determined in nine-month-old, male mice using Echo-MRI. Lean and fat mass was reduced in transgenic animals (*n* = 6). **i** Motor performance assessed in three independent tests was unchanged in three-month-old male ThyNAT mice (*n* ≥ 6). **j** PPI of the acoustic startle response was normal in 10-week-old, male ThyNAT mice (*n* = 5) compared with WT (*n* = 4). Data represent mean ± SEM. **p < 0.01; ***p < 0.001 compared with WT; Student’s *t* test

Our data indicated that neither lack of, nor excess NAA is tolerated. To investigate the cellular compartment most vulnerable to NAA toxicity, we generated transgenic animals with extra copies of the *Nat8l* gene under the neuronal Thy-1 promoter, in the *Aspa* WT background. Animals in this line (ThyNAT) were born according to Mendelian ratios and expressed the Flag epitope-tagged NAT8L protein specifically in the brain, but not in other organs (Fig. 3a). Histology revealed Flag-immunoreactivity specifically in the cytosol of CNS neurons (Fig. S4a). Numbers of thalamic neurons and oligodendrocytes were unchanged in ThyNAT compared with controls (Fig. S4b, c). A 1.5-fold *Nat8l* mRNA increase in brain lysates of ThyNAT mice compared with WT littermates was confirmed by qRT-PCR. *Rimkla/b* genes mediating NAAG synthesis and *Aspa* mRNA levels remained unchanged, indicating no substrate feed-forward inhibition at transcriptional level (Fig. 3b). Enhanced *Nat8l* mRNA expression translated in a 1.78 ± 0.07 and 1.47 ± 0.05 fold increase of NAA concentrations in cortical and brain stem extracts of ThyNAT mice (Fig. 3c). This was paralleled by depleted aspartate pools, reflecting increased incorporation into NAA. Osmolytes that were co-regulated with supra-physiological NAA levels in AKO, such as myo-inositol and taurine (Fig. 1h), were unchanged in ThyNAT mice. Myelination appeared normal and we did not observe vacuolization or astrocytosis (Fig. 3d). We found comparable NAAG concentrations in ThyNAT and controls (Fig. 3e) and unchanged amounts of the major myelin lipid galactosylceramide, further indicating normal myelination (Fig. 3f). Interestingly, we noticed a reduction in size and body weight starting at four weeks and persisting for at least one year (Fig. 3g). This was accompanied by a reduction in both lean and fat mass (Fig. 3h). Motor behaviour (Fig. 3i), startle amplitude (not shown) and sensorimotor gating (Fig. 3j) were normal in ThyNAT mice. The absolute cortical NAA levels in AKO correlated with tissue damage (as a function of neurotoxicity). In contrast, ThyNAT do not show signs of neurotoxicity even though they have higher absolute NAA levels in the cortex than AKO (Fig. S5). These results suggest that elevated NAA levels are uncoupled from neurological dysfunction and pathology.

### Selective Aspa deletion from oligodendrocytes causes Canavan disease

**Fig. 4:**
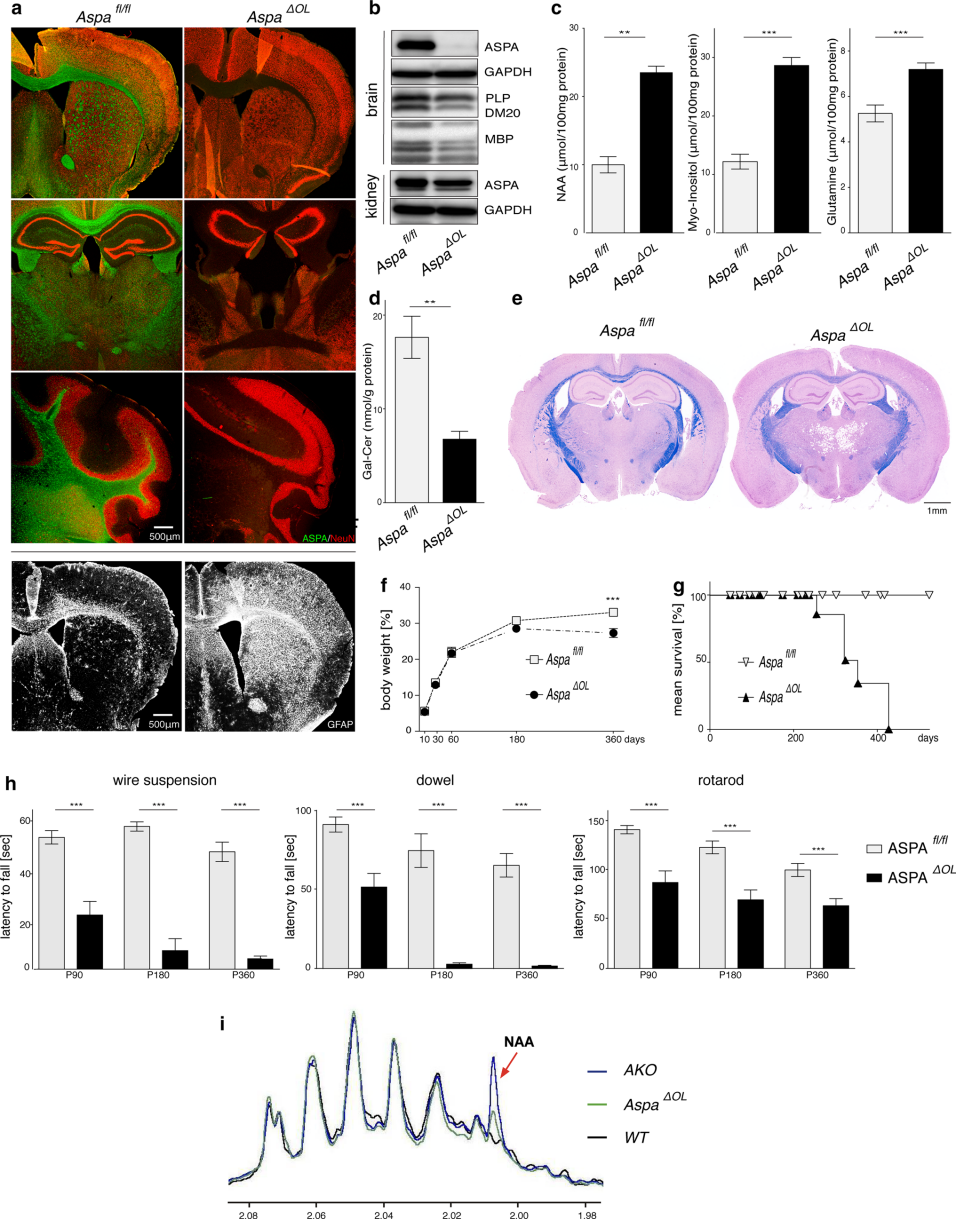
Selective ASPA depletion in oligodendrocytes causes CD with delayed progression. **a** Immunohistochemical staining for NeuN (red) and ASPA (green) in the striatum, thalamus and cerebellum of four-month-old *Aspa* ^*ΔOL*^ animals confirming the complete absence of ASPA throughout the CNS. GFAP immunohistochemistry revealed severe reactive astrogliosis in ASPA^ΔOL^ mice but not in controls. **b** Representative immunoblots showing that ASPA expression was abolished in protein lysates from six-month-old *Aspa* ^*ΔOL*^ brains (*n* = 3), but was preserved in the periphery. The central loss of ASPA is associated with reduced expression of the myelin markers PLP-1 and MBP. **c** Pool sizes of NAA, myo-inositol and glutamine were increased in cortex and brain stem of nine-month-old *Aspa* ^*ΔOL*^ mice (*n* = 10) compared with ASPA^fl/fl^ controls (*n* = 6). **d** Reduced galactosylceramide (Gal-Cer) concentrations were observed in the cortex of *Aspa* ^*ΔOL*^ mice (*n* ≥ 6). **e** H&E/LFB histology confirmed vacuolization and hypomyelination in 9-months-old *Aspa* ^*ΔOL*^ mice. **f** Body weight development in female *Aspa* ^*ΔOL*^ and *Aspa* ^*fl/fl*^ mice. Following normal development until 6 months of age the body weight of *Aspa* ^*ΔOL*^ mice plateaued and remained at reduced levels compared to controls (*n* = 5–18 per time point). **g** Reduced longevity of *Aspa* ^*ΔOL*^ mice. **h** Motor performance was assessed in the wire suspension test, the dowel test and the rotarod at three, six and nine months. Impaired motor skills of *Aspa* ^*ΔOL*^ mice (*n* = 4–14) compared with controls (*n* ≥ 8) were observed in all tests. Data represent mean ± SEM. **p < 0.01; ***p < 0.001; Student’s *t* test. **i** Representative ^1^H NMR spectra of kidney from *Aspa* ^*ΔOL*^, WT and AKO (*n* = 3). The arrowhead indicates the NAA peak at 2.01 ppm

Based on the lack of an overt molecular or cellular phenotype in ThyNAT mice in which NAA accumulation is restricted from oligodendrocytes due to normal ASPA function, we next investigated whether increasing NAA levels in the CNS, by deletion of the *Aspa* gene exclusively in oligodendrocytes, triggers a CD-like pathology. To address this, we developed mice with a conditional deletion of the *Aspa* gene exclusively in oligodendrocytes *(Aspa* ^*∆OL*^) by crossing 2**′**,3**′**-cyclic nucleotide 3**′** phosphodiesterase (Cnp) Cre recombinase knock-in mice (*CNP*-*Cre*) with conditional-ready mutant mice in which the second exon of the *Aspa* gene was flanked by loxP Cre binding sites (*Aspa* ^*fl/fl*^). *Aspa* ^*∆OL*^ mice were born at the expected Mendelian ratio. Histological examination revealed that ASPA-immunoreactivity was completely abolished in the *Aspa* ^*∆OL*^ brain. There was no compensatory ectopic regulation in any other cell type in the CNS (Fig. 4a). Widespread astrogliosis even in the striatum, a brain region not affected by vacuolization, indicated pathology. Immunoblot analysis confirmed that oligodendroglial *Aspa* deletion in *Aspa* ^*∆OL*^ mice abolished ASPA expression throughout the brain while ASPA expression in peripheral organs, including the kidney, remained intact. PLP and MBP protein levels were reduced in *Aspa* ^*∆OL*^ mice, suggesting demyelination (Fig. 4b). Quantification of metabolite pools in the brainstem of *Aspa* ^*∆OL*^ mice showed a marked increase in NAA, myo-inositol and glutamine (Fig. 4c). Hypomyelination was confirmed by reduced galactosylceramide amounts in brain homogenates (Fig. 4d) and reduced myelin staining in histological sections (Fig. 4e). In the thalamus of *Aspa* ^*∆OL*^ mice, a brain region showing pronounced vacuolization, numbers of both neurons and oligodendrocytes were reduced compared with *Aspa* ^*fl/fl*^ (Fig. S6). In comparison to age-matched AKO mice (Fig. S2d)*, Aspa* ^*∆OL*^ animals showed relatively moderate vacuolization, even at nine months of age. The physical development of *Aspa* ^*∆OL*^ mutants assessed by body weight monitoring was normal up to six months, but was followed by attenuated weight gain compared to age-matched controls (Fig. 4f). Matching our observation in the AKO line (Fig. 1e), longevity of *Aspa* ^*∆OL*^ mice was limited to 14 months. However, we did not observe premature fatalities around three weeks of age (Fig. 4g) suggesting a role of peripheral ASPA for juvenile survival. Motor performance, tested between three months and one year, was progressively impaired in *Aspa* ^*∆OL*^ compared with controls (Fig. 4h). Compared with AKO, severity of motor deficits in *Aspa* ^*∆OL*^ mice was milder up to six months but converged afterwards when *Aspa* ^*∆OL*^ mice also presented with paralysis and seizures. NAA levels in the kidney were below the detection limit in WT or *Aspa* ^*f/f*^ controls and were detectable at low levels in *Aspa* ^*∆OL*^ samples (Fig. 4i). In contrast, benchmark AKO samples showed a substantial NAA peak. These findings provide evidence that centrally derived NAA can be degraded by ASPA expressed in peripheral organs. In summary, compared with the whole body knockout, ablating ASPA in oligodendrocytes results in a CD model with delayed progression and partially attenuated late-stage severity.

### Preclinical CD gene therapy targeted to symptomatic oligodendrocytes

The data we obtained by phenotyping different CD mouse models showed that oligodendroglial ASPA acts as a natural safeguard against NAA toxicity in the CNS. Next we aimed at translation of our findings for development of a preclinical CD gene therapy based on *ASPA* gene delivery to oligodendrocytes. As a result of characterizing early disease progression in AKO mice, we identified that supra-physiological levels of NAA and other metabolites, brain vacuolization and hypomyelination manifested at p30 (Fig. S7). To achieve clinical relevance we selected this symptomatic stage as time point of intervention.

**Fig. 5:**
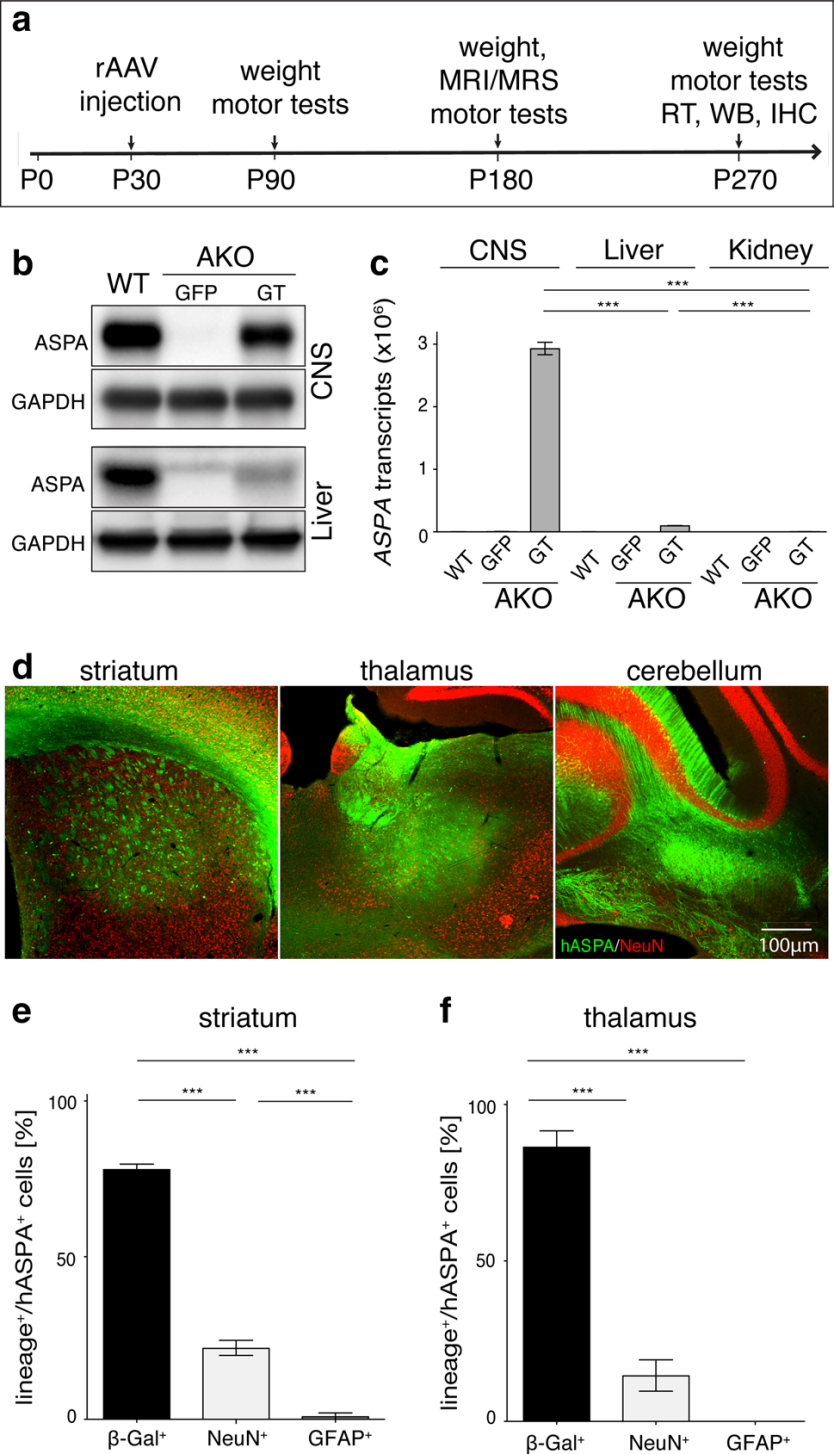
Long-term oligodendroglial *ASPA* expression following post-symptomatic AAV delivery. **a** Schematic overview of the preclinical cy5-*Mbp*-*ASPA* gene replacement therapy. **b** Immunoblot confirming stable recombinant *ASPA* expression in the CNS of AKO-GT (AAV-*Mbp*-*ASPA*) mice compared to AKO-GFP (AAV-*Mbp*-GFP) control mice at 9 month of age and virtually no expression in the AKO liver following intracranial therapeutic AAV delivery. **c** qRT-PCR detection of human *ASPA* mRNA expression in the CNS of nine-month-old cy5-*Mbp*-*ASPA* treated AKO mice. Transgenic *ASPA* mRNA expression was minimal in the liver and kidney. **d** Immunohistochemical detection of vector spread achieved by intracranial delivery of 2 × 10^9^ vg cy5-*Mbp*-*ASPA* to the striatum, thalamus and cerebellum of P30 AKO mice. Percentage of β-Gal^+^ oligodendrocytes, NeuN^+^ neurons and GFAP^+^ astrocytes expressing transgenic human ASPA (hASPA) in the **e** striatum and the **f** thalamus of AKO mice eight months following AAV delivery (*n* = 3). Data represent mean ± SEM. ***p < 0.001; One-way ANOVA with Holm-Sidak post hoc test

We have previously shown that driving oligodendrocyte-specific transgene expression in the CNS is possible using the recombinant mouse myelin basic protein promoter (*Mbp*) in adeno-associated viral (AAV) vectors [29, 80]. We applied this platform to deliver the human ASPA cDNA (GT mice) or green fluorescent protein (GFP) by direct multi-site injections to AKO followed by longitudinal phenotyping (Fig. 5a). Immunoblotting and qRT-PCR confirmed stable and abundant expression of transgenic ASPA in the CNS of GT mice (Fig. 5b, c). In contrast, virtually no expression of transgenic ASPA was observed in peripheral organs. Immunohistochemical staining corroborated ASPA expression in all target regions and a robust vector spread (Fig. 5d) comparable to our previously published results [80]. Identification of transgene expressing cells in the target areas confirmed approximately 80% oligodendrocyte specificity in the striatum and thalamus alike (Fig. 5e, f; Fig. S8) indicating that at least early during the CD pathology specificity of transgene expression and transduction efficiency does not depend on the tissue integrity at the time point of vector delivery. While the cerebellum was not analysed quantitatively, we approximated equal numbers of hASPA expressing neurons and oligodendrocytes in this region. ASPA was detected in oligodendrocytes of myelinated tracts such as the corpus callosum and fimbria suggesting penetration of the virus vector into compact white matter (not shown).

### Post-symptomatic oligodendroglial ASPA gene therapy reverts Canavan disease

**Fig. 6:**
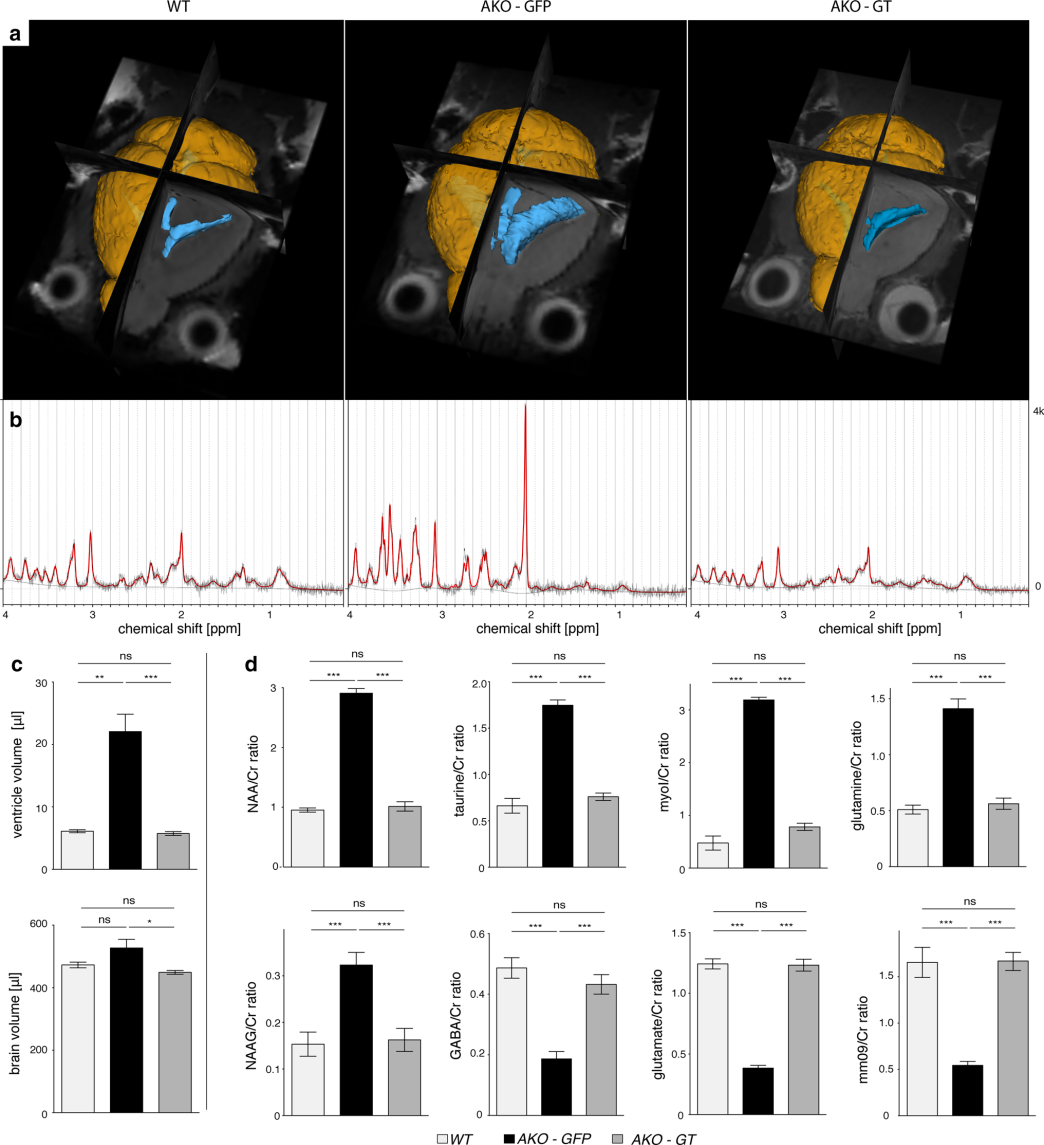
AAV-*Mbp*-*ASPA*-mediated gene therapy in AKO oligodendrocytes restores NAA levels and brain size. **a** Representative 3D reconstruction of T2-weighted MRIs from male WT, AKO-GFP and AKO-GT mice. Segmentation of images was performed in 3D Slicer outlining brain surface (yellow) and ventricles (blue). **b** Representative traces of ^1^H-MR spectroscopy obtained from the above animals. **c** Normalized brain volume and ventricle size obtained from reconstructed T2-weighted MRIs in AKO-GT animals compared to AKO-GFP controls. **d** ^1^H-MR spectroscopy-based quantification of metabolite pools relative to total creatine in the brain stem from six-month-old WT, AKO-GFP, and AKO-GT mice (*n* = 3). Note, the mm09 refers to a macromolecular and lipid fraction with chemical shift of 0.9 ppm. Data represent mean ± SEM. *p < 0.05; **p < 0.01; ***p < 0.001; One-way ANOVA with Holm-Sidak post hoc test

**Fig. 7:**
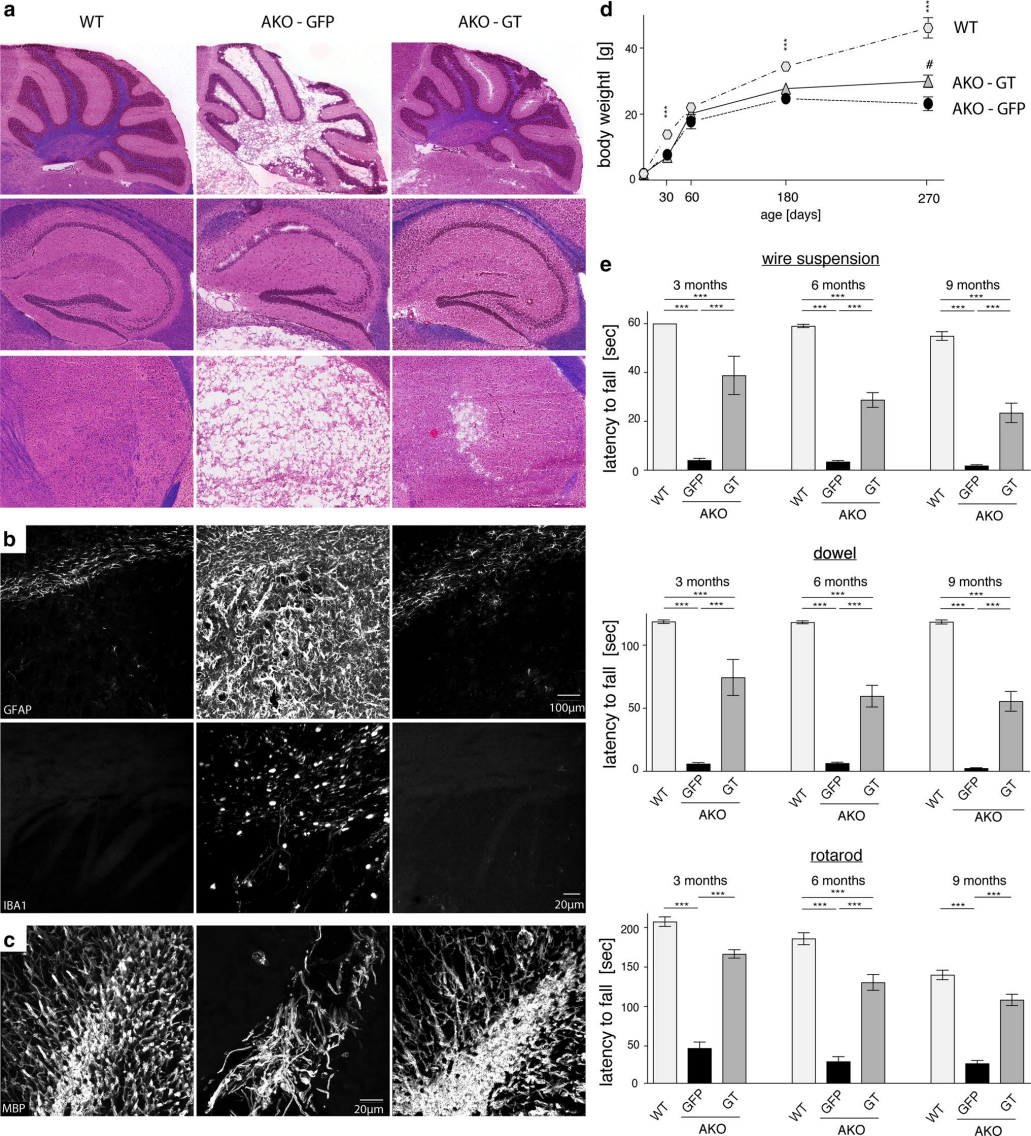
Post-symptomatic intracranial gene therapy targeting oligodendrocytes improves histopathology and motor performance in CD mice at nine months. **a** Representative H&E/LFB histology of the cerebellum, hippocampus, thalamus and striatum (from top to bottom) of WT, AKO-GFP, AKO-GT animals. **b** Representative immunohistochemical staining in the striatum showing normalization of astrogliosis (GFAP; top) and microgliosis (IBA1; bottom) in GT-treated mice. **c** ASPA gene therapy improves myelination assessed by normalized MBP immunoreactivity in the cerebellum. **d** Attenuation of weight of aged AKO following intracranial cy5-*Mbp*-*ASPA* gene therapy. **e** Motor performance was assessed at three, six and nine months. Sustained improvement in the wire suspension test, dowel test and on the rotarod approaching WT levels was measured in AKO-GT animals compared to GFP controls (*n* = 4–30). Data represent mean ± SEM. **p < 0.01; ***p < 0.001; #*p* < 0.05 (AKO-GFP vs AKO-GT). One-way ANOVA with Holm-Sidak post hoc test

High resolution T_2_-weighted MRI 5 months following intracranial *Mbp*-*ASPA* gene therapy revealed that key aspects of the CD histopathology were reverted in AKO-GT mice (Fig. 6a). Representative in vivo ^1^H-MR spectra measured in the thalamus of these animals indicated normalisation of the metabolic profile (Fig. 6b). Quantification of brain and ventricle volume revealed that GT normalized both brain volume and excessive ventricle dilation (Fig. 6c). Quantitative analysis of ^1^H-MR spectra confirmed restoration of excessive NAA and NAAG concentration, normalization of organic metabolite levels exemplified by myo-inositol, taurine and the macromolecular and lipid fraction, as well as replenishment of the glutamate and GABA neurotransmitter pools (Fig. 6d). Neurochemical restoration in GT mice was corroborated by attenuation of brain damage (Fig. 7a). Compared to AKO-GFP controls, in AKO-GT mice numbers of both neurons and oligodendrocytes were restored in the thalamus, a target site for AAV-ASPA delivery (Fig. S9). Immunohistochemical assessment affirmed absence of reactive astrogliosis and microgliosis in AKO-GT mice (Fig. 7b), as well as improved myelination (Fig. 7c). We repeatedly assessed locomotor behaviour employing hanging wire, dowel and rotarod tests and found long-term improvements in GT mice (Fig. 7d).

## Discussion

### Lack of NAA causes neurological dysfunction

Recent studies have indicated that *Nat8l* could be a therapeutic target for the treatment of CD. This concept prompted a detailed investigation of the consequences associated with altered *Nat8l* expression in vivo. As expected*, Nat8l*-deficiency in NKO and DKO mice resulted in the complete loss of NAA and its derivative NAAG. This indicates that NAA, while likely to contribute to osmoregulation, myelination, energy and pH homeostasis in the brain [56, 66, 68, 77], is not essential for these processes. However, when employing tests of sensorimotor function and neuronal recruitment, we identified hitherto unreported deficits in NAA-deficient lines. The notable reduction in the auditory startle response despite normal motor behaviour and functional auditory processing indicates a possible role for NAA in the limbic pathways protecting the body from predatory attacks or sudden trauma. Researching the exact underlying course of action by which NAA-deficiency causes reduced neuronal fibre recruitment following audio-visual stimulation and ASR attenuation was outside the scope of this study. However, our data suggest that aberrant aspartate- or NAAG-dependent neuromodulation may underlie dysfunction at the neurocircuitry level. The increased aspartate pool size resulting from absence of NAT8L can be explained by substrate accumulation. This shift may affect the malate–aspartate shuttle causing alterations in subcellular glutamate localisation and neuronal energy homeostasis. In fact, lack of the neuronal aspartate–glutamate carrier is associated with hyperreflexia [82]. In addition, aspartate can influence the excitatory function of GABAergic cells and promote GABA release via the selective activation of NMDA receptors [43]. Imbalances of GABAergic and glycinergic neurotransmission in axons of giant neurons of the pontine reticular formation, as well as loss of these cells have been associated with impaired ASR [41], and NMDA receptor activity is critical for a normal ASR response [37]. Attenuated PPI, observed in the NKO and DKO lines, has been linked to neuropsychiatric disorders including schizophrenia. These sensorimotor gating deficits may be caused by dysregulation of the dopamine system which has previously been reported for* Nat8* *l*-deficient mice [41, 62, 73].

### Elevated NAA concentrations are not intrinsically neurotoxic

Absence of ASPA, causing a detrimental increase of the organic metabolite NAA within oligodendrocytes is the root cause for the neurochemical changes, histopathology and motor deficits observed in CD. Utilizing alternative models, we confirm that key aspects of the pathology are paralleled by dose-dependent NAA toxicity and that NAA depletion prevents CD [32, 51]. Metabolic profiling disclosed a substantial increase in principal osmolytes in *Aspa* depleted mice that was proportional to the observed spongiform vacuolisation, ventricle size and macrocephaly. In the brain, cell volume has been proposed to be predominantly regulated by organic osmolytes because their flux, in contrast to ions, does not directly perturb the membrane potential and thus electrical activity [5]. Pools of these osmolytes are primarily located in glia and are directly involved in the regulation of water homeostasis, membrane stability, pH and survival [8, 14, 16, 21, 23, 34]. NAA distribution is dynamically regulated during osmotic fluctuations, but the role of NAA as osmolyte remains controversial [6, 8, 18, 30, 64, 72, 74, 76]. Importantly, our findings indicate that increased NAA concentrations are uncoupled from CNS pathology and neurological dysfunction. Direct comparison of cortical NAA elevation to equivalent levels either by neuronal NAT8L overexpression (ThyNAT), or *Aspa* deletion, provides evidence that NAA toxicity depends on the lack of its oligodendroglial degradation. This suggests that intact ASPA in oligodendrocytes of ThyNAT mice maintains oligodendrocyte NAA at physiological concentrations both intracellularly and in the myelin compartment, thereby preventing disease. Consequently, the increased overall NAA levels in ThyNAT brain extracts reflect increased extracellular or intra-neuronal concentrations that are not toxic per se and do not trigger a substantial alteration in principal osmolytes. This is supported by recent findings that extracellular elevation of NAA does not cause vacuolating histopathology [4]. While the aetiology is multifactorial, based on our data, we hypothesize that the CD pathology is triggered by oligodendroglial intolerance to supra-physiological NAA concentrations. Increased NAA may be particularly detrimental to oligodendrocytes by inducing osmotic challenges to a cell designated to maintain a highly lipid-rich environment or by inducing oxidative stress and lipid peroxidation [24]. Additionally, challenges to maintain energy homeostasis might contribute to the aetiology. NAA-derived aspartate is metabolised by oligodendrocytes where it is thought to contribute to the import of reducing equivalents into mitochondria and enter the tricarboxylic acid cycle [1]. Because NAA is not metabolised, AKO oligodendrocytes might thus experience sustained dysregulation of energy homeostasis. Irrespective of whether oligodendroglial NAA toxicity, lipid peroxidation, or challenges maintaining intracellular osmotic or energy homeostasis are the root cause, the CD pathology is exacerbated by a secondary increase of organic osmolytes involving astrocytes.

While this study confirms that NAA is not essential for normal myelination, the incorporation of NAA-derived acetate into myelin lipids and thus its contribution to myelin synthesis is undisputed [9, 13, 17, 32, 49, 51, 53, 81]. The complex interplay between astrocytes, oligodendrocytes and neurons is only beginning to be understood. In fact, astrocytes have recently been shown to contribute substantial amounts of lipid to oligodendrocytes during myelination [11]. Our findings suggest that oligodendrocytes, that synthesize and preserve a highly lipid-rich environment in the brain, are more involved in osmolyte and energy homeostasis than previously anticipated.

### Peripheral ASPA is implicated in juvenile survival

Despite normal survival and the absence of CNS abnormalities, we observed substantial growth retardation in ThyNAT mice that mirrors a peripheral aspect of the pathology observed in AKO and CD. Recent reports have implicated NAA in the energy regulation of peripheral tissues [59, 71]. While not assessed in this study, export of neuron-derived recombinant NAA affecting adipocyte metabolism might be responsible for the observed growth retardation in ThyNAT mice. However, given our investigations were limited to one transgenic line, non-specific position effects affecting physical development cannot be excluded. The virtually normal body weight of *Aspa* ^*∆OL*^ mice vs. the diminished body weight of AKO may suggest a role of peripheral ASPA and/or excess NAA for maintaining adipose tissue and lipid turnover. In fact, our findings are in agreement with a report showing that increased NAA, especially in conjunction with reduced ASPA activity, prevents brown adipose tissue cells from expressing mature markers [59].

Disrupted *Aspa* gene function in AKO and DKO was associated with a biphasic impairment illustrated by a first wave of premature death around four weeks of age. Early phase survivors continued to live until approximately 14 months. These findings match data recently obtained from independent DKO lines [51]. Our data suggest that premature death, observed in DKO and AKO alike, is caused by *Aspa* deletion, as NKO mice showed normal survival rates. Comparable to DKO and AKO, longevity of *Aspa* ^*∆OL*^ mice was reduced indicating that central ASPA dictates long-term survival. These conditional ASPA mutants, lacking central but not peripheral ASPA, showed normal juvenile survival, indicating loss of ASPA expression outside the CNS renders young animals vulnerable to early detrimental events. Furthermore, normal numbers of oligodendrocytes and neurons in DKO underline that impaired survival in this line may have a peripheral origin. AKO and *Aspa* ^∆OL^ showed substantial loss of both lineage cells. These data show that cell loss is not limited to oligodendrocytes and that NAA depletion prevents cell death, confirming reports in other CD mouse models [44, 69]. Our data do not inform, however, if the loss of one cell lineage precedes demise of the other. The results of our comprehensive phenotypic characterization of NAA-mutant mouse lines are summarized in Table S1. These data have important therapeutic implications as they indicate that even peripheral enzyme replacement therapies could directly benefit CD patients particularly during early stages of the disease.

### Aspartoacylase expression in peripheral organs ameliorates Canavan disease

*Aspa* ^*∆OL*^ mice, that lack *Aspa* expression exclusively in oligodendrocytes, were devoid of ASPA-immunoreactivity in the entire CNS, while *Aspa* expression in peripheral organs was normal. Onset of CD histopathology and behavioural deficiencies were significantly delayed. Despite the fact that *Nat8l* production was unaltered in these animals, NAA levels in the CNS were markedly lower than in AKO mice. These data indicate that peripheral ASPA functions as a metabolic sink for excess NAA draining off from the CNS. While immunological impairments and decreased macrophage survival have recently been reported in ASPA-null mice [3, 28], the CD pathology is virtually exclusively confined to the CNS. Consequently efforts for development of an enzyme replacement therapy focussed on development of *ASPA* modification that would allow the recombinant enzyme to cross the blood–brain barrier [58].

### Post-symptomatic oligodendroglial ASPA gene therapy reverts Canavan disease pathology

CD is a bona fide target for gene therapy as it is a devastating, fatal childhood disorder and there is no treatment available. In the mouse, a naturally short-lived mammalian species, systemic AAV-mediated *ASPA* delivery has recently shown great therapeutic success [2, 30]. Nevertheless, immunogenicity and limitations in production continue to impede systemic AAV delivery for treatment of CNS disorders in humans [42]. The CNS is widely recognized as an immune privileged organ as it largely lacks a potent innate immune response [20]. Importantly, direct intracranial AAV-mediated *ASPA* delivery to CD patients has already proven safe in the clinic [48].

We demonstrated that in the CNS, *Aspa* is exclusively expressed in oligodendrocytes and that these cells are at the root of CD pathology. We thus hypothesize that complete and long-term therapeutic benefit will be achieved best by restoring ASPA expression in oligodendrocytes, the cell type that naturally expresses the enzyme in the CNS. In line with this hypothesis direct intracranial AAV-*ASPA* gene delivery to CNS neurons showed limited therapeutic benefit [2, 3, 40], while in a recent study neonatal delivery of a novel AAV serotype that preferably infects oligodendrocytes prevented development of CD in ASPA^*nur7/nur7*^ mice, at least until three months of age [25]. In the vast majority of cases, onset of pathology and associated symptoms precede diagnosis. Subsequent gene therapy treatment is likely to be delayed even further. To address this, we aimed to treat AKO mice following manifestation of CD pathology. Based on our previous finding that AAV-mediated transgene expression can be restricted to oligodendrocytes by utilizing oligodendroglial promoters [29, 78, 80] we employed a post-symptomatic *Mbp*-*ASPA* gene therapy in AKO mice at P30. Due to its weak immunogenicity, good packaging capacity and infectivity, we utilized cy5, a variant of AAV7 that has been recognized for its potential as clinical gene therapy vector [27]. We achieved oligodendroglial transgenic *ASPA* expression beyond 9 months of age resulting in normalization of NAA and metabolite concentrations, restoration of CNS pathology present at the time point of injection and neurological amelioration. While gene therapy worked best in young CD patients [48], our preclinical data suggest the presence of a therapeutic window that may be relevant for future clinical studies. However, our post-symptomatic intracranial injection improved, but did not rescue the relative lag of body weight in AKO indicating a peripheral contribution to this phenotype in the CD pathology. The good overall therapeutic outcome would likely to be improved by combining intraparenchymal injections with intrathecal and intracerebroventricular delivery to improve therapeutic ASPA expression in the dorsal brainstem and spinal cord. As the brainstem is involved in the control of vital body functions, direct injection into the brainstem was avoided in this study. Both the intrathecal and intracerebroventricular delivery routes are already successfully performed in clinical trials and well tolerated. In addition, lowering NAA burden through peripheral enzyme replacement therapy, *Nat8l* knockdown, or via creating an intracranial metabolic sink by ectopic astroglial ASPA expression as proposed by the Gao lab should be considered as a complementary therapeutic strategy [31]. Taken together, this study advances the knowledge around the role of NAA in health and disease and uncovers a feasible roadmap to the successful treatment of CD and other myelin disorders.

## Electronic supplementary material

Below is the link to the electronic supplementary material.

Supplementary material 1 (PDF 6902 kb)

## Abbreviations

AAV: Adeno-associated virus
ASPA: Aspartoacylase
CD: Canavan disease
NAT8L: *N*-Acetyltransferase 8-like
NAA: *N*-Acetylaspartate
NAAG: *N*-Acetylaspartylglutamate
AKO: *Aspa* knockout
NKO: *Nat8l* knockout
DKO: *Nat8l/Aspa* double-knockout
ThyNAT: Thy-*Nat8l* transgenic mice
*Aspa*^*∆OL*^: Conditional oligodendrocyte-specific *Aspa* knockout
N^+/−^AKO: *Aspa* knockout with one deleted *Nat8l* allele
AKO-GT: ASPA knockout subjected to gene therapy with AAV-ASPA
AKO-GFP: ASPA knockout treated with AAV-GFP control vector
