# Supplementary material for: Uncoupling N-acetylaspartate from brain pathology: implications for Canavan disease gene therapy
Source: Acta Neuropathol. 2017 Nov 7;135(1):95–113. doi: 10.1007/s00401-017-1784-9 (PMC5756261; doi:10.1007/s00401-017-1784-9)
Supplement: Supplementary file 1 — Supplementary material 1 (PDF 6902 kb) [file 401_2017_1784_MOESM1_ESM.pdf]

Supplementary Material

Uncoupling N-acetylaspartate from brain pathology - Implications for Canavan disease gene therapy

Georg von Jonquieres[1#], Ziggy H. T. Spencer[1], Benjamin D. Rowlands[1,2], Claudia B. Klugmann[1], Andre Bongers[3], Anne E. Harasta[1], Kristina E. Parley[1], Jennie Cederholm[1], Orla Teahan[1], Russell Pickford[4], Fabien Delerue[5], Lars M. Ittner[2,5,6], Dominik Fröhlich[1], Catriona A. McLean[7], Anthony S. Don[8], Miriam Schneider[9], Gary D. Housley[1], Caroline D. Rae[2], Matthias Klugmann[1#]

#Correspondence should be addressed to Matthias Klugmann or Georg von Jonquieres, Translational Neuroscience Facility, School of Medical Sciences, UNSW, Sydney, Australia. Phone: +61-2-93851056. E-mail: m.klugmann@unsw.edu.au or g.jonquieres@unsw.edu.au

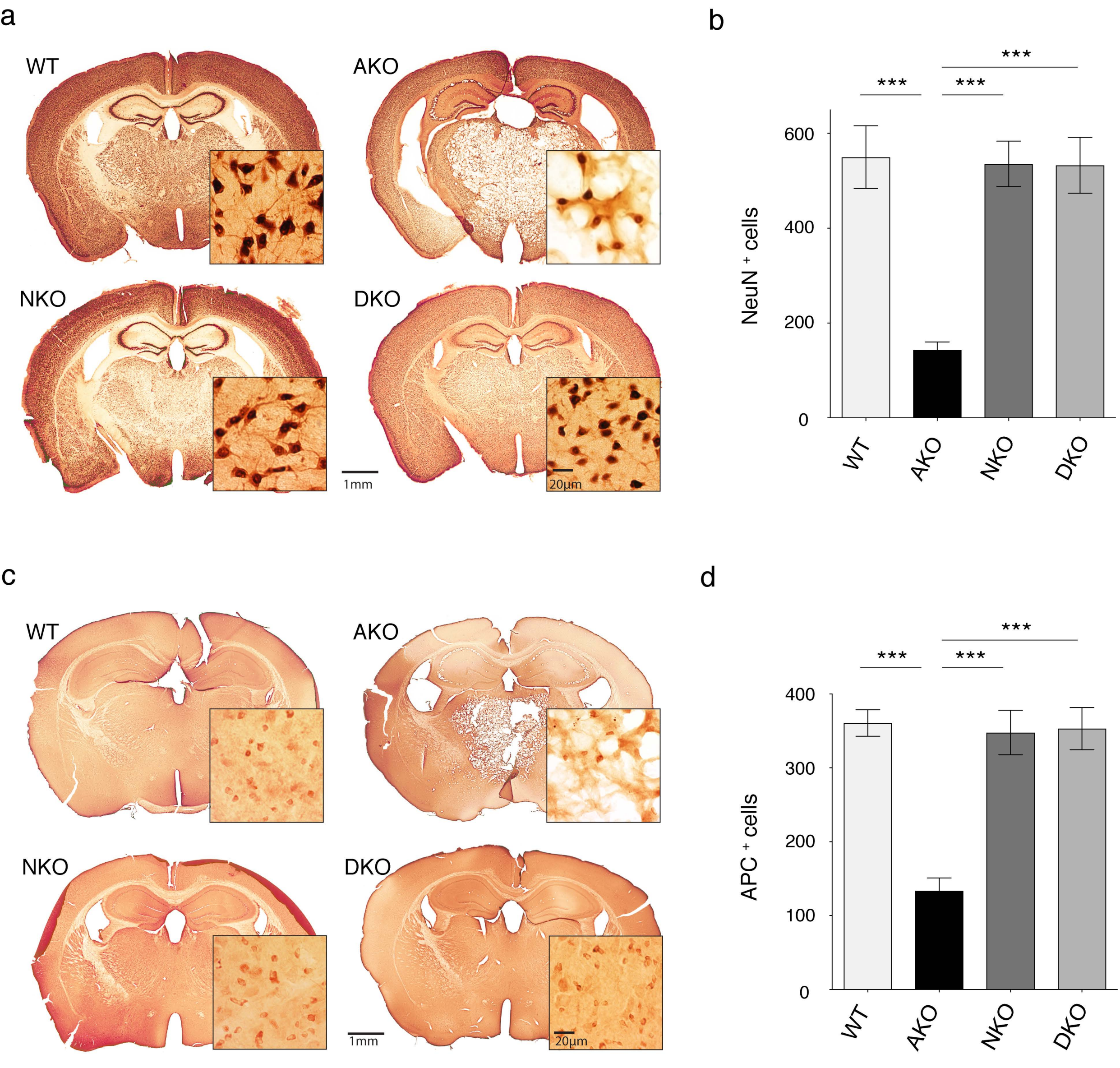
a
WT
AKO
NKO
DKO
1mm
20μm
b
***
***
***
NeuN + cells
600
400
200
0
WT
AKO
NKO
DKO
c
WT
AKO
NKO
DKO
1mm
20μm
d
***
***
***
APC + cells
400
300
200
100
0
WT
AKO
NKO
DKO

Fig. S1

Loss of both neurons and oligodendrocytes in the thalamus of AKO mice is rescued by NAA depletion. Immunoperoxidase detection of **a** NeuN as marker for neurons and **c** APC as marker for oligodendrocytes in coronal sections of nine month old WT, AKO, NKO and DKO mice. Each Diamminobenzidine (DAB) stained overview section is complemented by a high magnification inset and a separate dashed square approximating the region devoted to quantification of lineage positive cells. Graphs visualising the average number of **b** NeuN+ or **d** APC cells in a 300 μm^2 section of the thalamus (n=3). Data represent mean ± SEM. ***$p < 0.001$; One-way ANOVA with Holm-Sidak *post hoc* test.

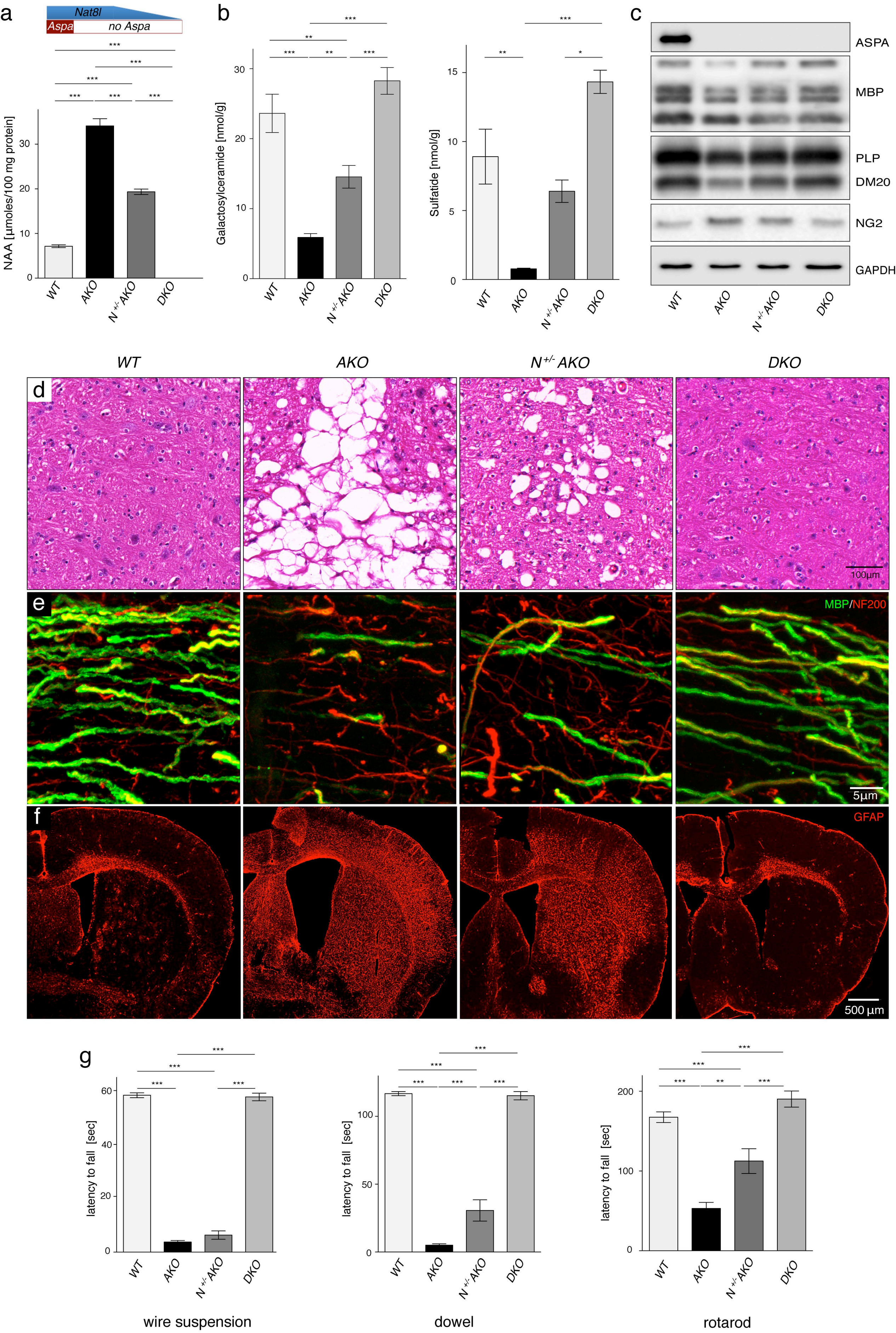

a
Nat8l
Aspa
no Aspa
NAA [µmoles/100 mg protein]
WT
AKO
N+/- AKO
DKO
b
Galactosylceramide [nmol/g]
Sulfatide [nmol/g]
c
ASPA
MBP
PLP
DM20
NG2
GAPDH
WT
AKO
N+/- AKO
DKO
d
100µm
e
MBP/NF200
5µm
f
GFAP
500 µm
g
latency to fall [sec]
wire suspension
dowel
rotarod

Fig. S2

Genetic titration of NAA progressively improves the CD-like pathology in AKO mice. **a** Quantification of NAA concentrations in cerebellar extracts of AKO mice ($n \geq 4$) with full, halved, or abolished *Nat8l* gene dosage. In the absence of its enzymatic degradation, NAA produced by one or two *Nat8l* gene copies results in approximately twice or four times the amount of NAA compared with controls. **b** Levels of galactosylceramide and sulfatide are gradually restored upon reduction of the *Nat8l* gene dose in AKO mice ($n \geq 5$). **c** Representative immunoblots displaying the absence of ASPA in the CNS of AKO, $N^{+/-}$AKO and DKO mice ($n = 3$) show restoration of MBP and PLP levels compared to GAPDH following gradual reduction of the *Nat8l* gene dose. In contrast the increased NG2 expression in AKO is diminished in $N^{+/-}$AKO mice and reaches WT expression levels when both ASPA and NAT8L is depleted in DKO mice. **d** High power microscopy images of coronal sections from six months old WT, AKO, $N^{+/-}$AKO and DKO mice, stained with H&E to visualize gross tissue morphology and myelination. Lowering the *Nat8l* gene dose progressively in the AKO background ameliorates brain damage reflected by hypomyelination, vacuolization and ventricle dilation. **e** Representative high power confocal images of superficial cortical layers that are not affected by vacuolisation immunostained for MBP (green) and NF200 (red) in six months old WT, AKO, $N^{+/-}$AKO and DKO mice. Loss of myelin in AKO is restored in DKO. **f** Immunohistochemical staining for GFAP in striatal brain sections from WT, AKO, $N^{+/-}$AKO and DKO. **g** Gradual reduction of the *Nat8l* gene dose improves motor deficits observed in AKO mice (tested at six months). Data represent mean ± SEM. $^{**}p < 0.01$; $^{***}p < 0.001$; One-way ANOVA.

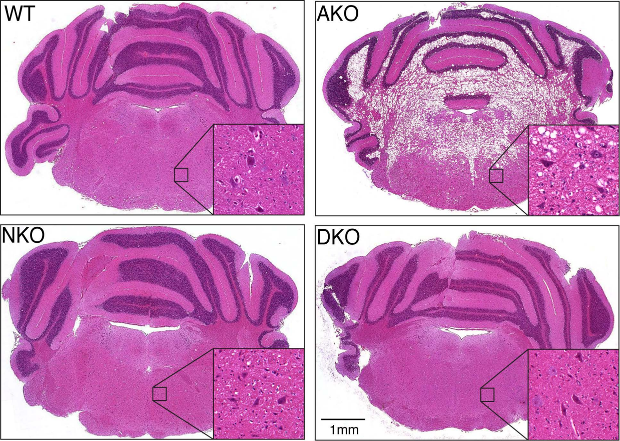
WT
AKO
NKO
DKO
1mm

Fig. S3:

Structural integrity of the PnC in NAA-mutant mice. H&E stained sections illustrate integrity of the ventral brain stem in WT, AKO, NKO and DKO at six months. Inset: Giant neurons in the caudal pontine reticular nucleus (PnC).

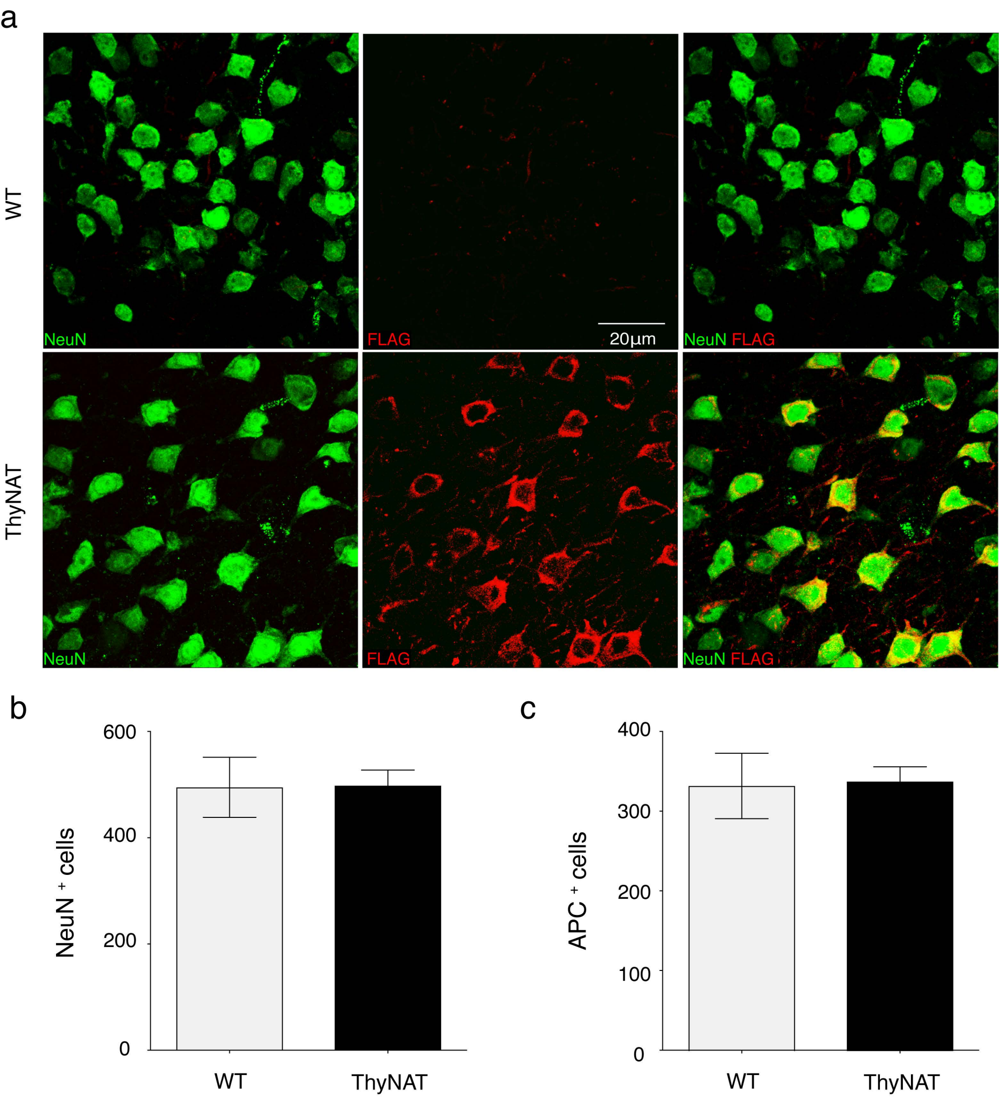
a
WT
ThyNAT
NeuN
FLAG
20µm
NeuN FLAG
b
NeuN + cells
600
400
200
0
WT
ThyNAT
c
APC + cells
400
300
200
100
0
WT
ThyNAT

Fig. S4

Transgenic NAT8L expression is restricted to neurons. **a** Representative histological images showing Flag-immunoreactivity (red) in cortical neurons (detected by NeuN, green) of a three-month old ThyNAT animal (bottom row) but not in the control (top row). Note the cytosolic cellular localisation of transgenic NAT8L. **b** Quantification of immunoperoxidase stained neurons ($NeuN^+$ cells) and oligodendrocytes (APC^+ cells) in the thalamus of nine months old WT and ThyNAT mice ($n = 3$). Data represent mean ± SEM ; One-way ANOVA.

a

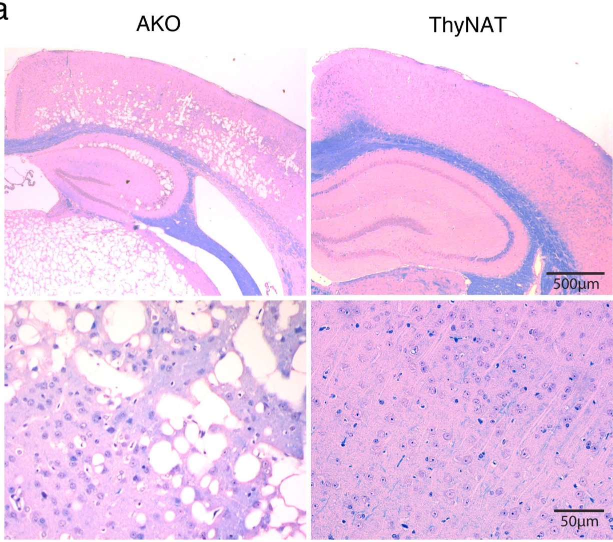
AKO
ThyNAT
500μm
50μm

b

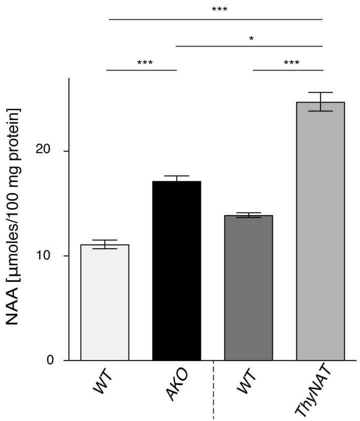
***
*
***
***
NAA [μmoles/100 mg protein]
0
10
20
WT
AKO
WT
ThyNAT

Fig. S5

Neurotoxicity is uncoupled from supraphysiological NAA. **a** Representative histological images showing H&E / Luxol Fast Blue stained cortical sections of ThyNAT and AKO at 9 months of age. Note the vacuoles in the AKO cortex. **b** NAA levels in the cortex of ThyNAT, AKO and corresponding WT controls (n = 3). NAA data from ThyNAT and WT are from Fig. 3c. Data represent mean ± SEM ; One-way ANOVA.

b

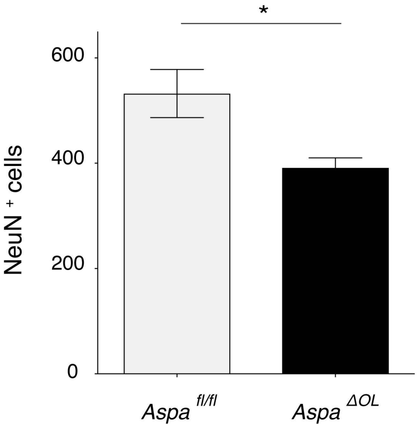
*
600
400
200
0
NeuN + cells
Aspa fl/fl
Aspa ΔOL

c

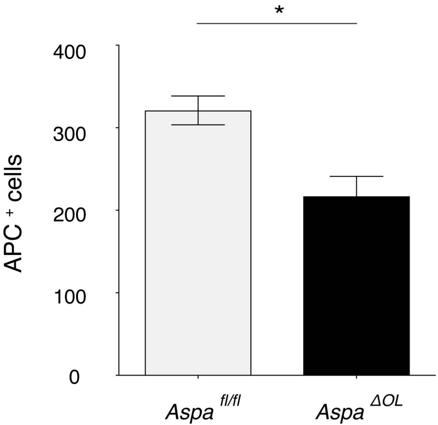
*
400
300
200
100
0
APC + cells
Aspa fl/fl
Aspa ΔOL

Fig. S6

Oligodendroglial ASPA depletion is associated with loss of neurons and oligodendrocytes alike. Coronal sections of *Aspa*$^{\Delta OL}$ and *Aspa*$^{fl/fl}$ mice were immunoperoxidase stained with **a** NeuN as marker for neurons or **b** APC as marker for oligodendrocytes. Lineage positive cells in 300 μm^2 sections of the thalamus were counted and graphed ($n = 3$). Data represent mean ± SEM. *$p < 0.05$; One-way ANOVA with Holm-Sidak *post hoc* test.

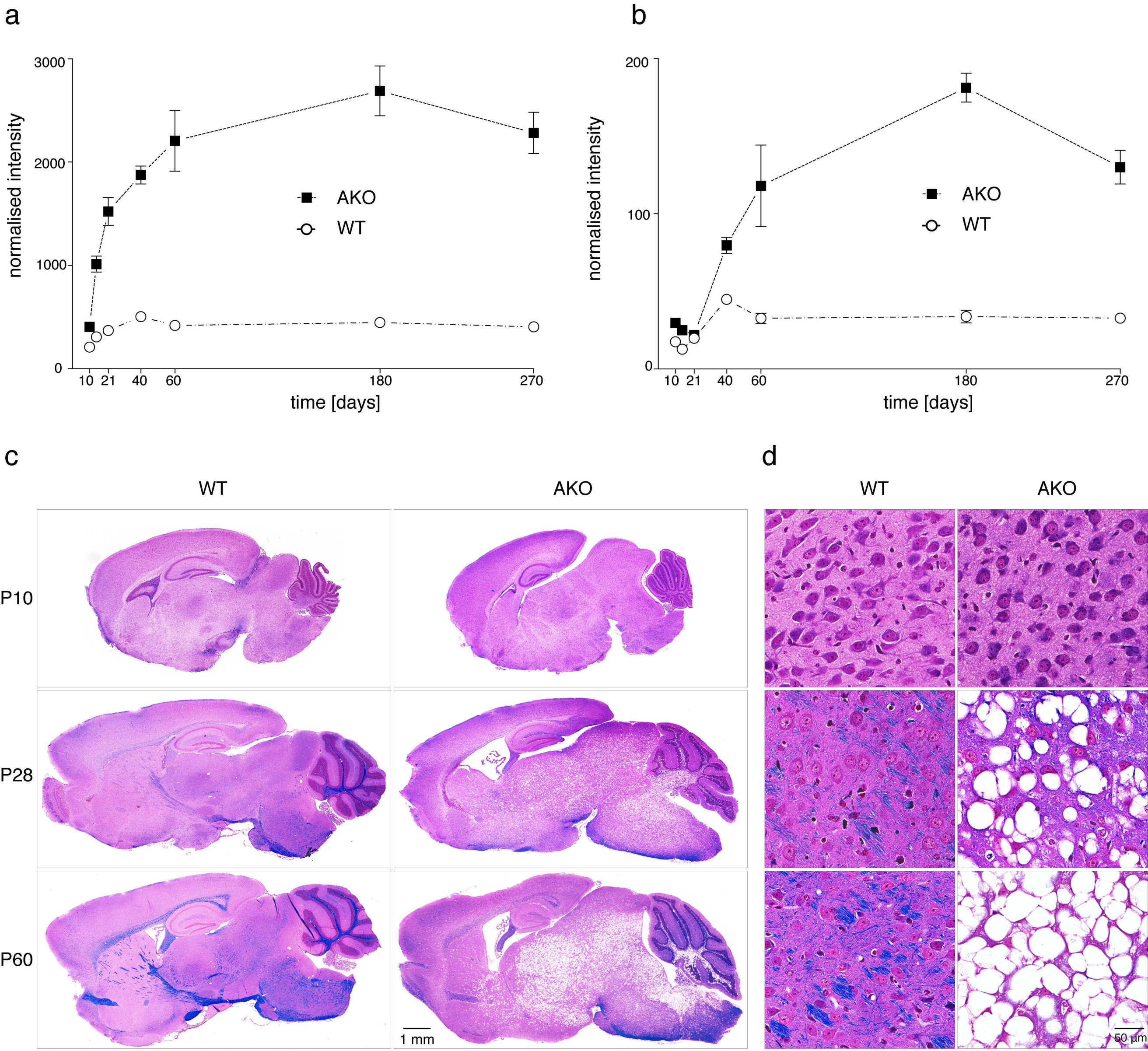
a
3000
2000
1000
0
normalised intensity
AKO
WT
10
21
40
60
180
270
time [days]
b
200
100
0
normalised intensity
AKO
WT
10
21
40
60
180
270
time [days]
c
WT
AKO
P10
P28
P60
1 mm
d
WT
AKO
50 µm

Fig. S7

Onset of CD in AKO mice. **a** Longitudinal development of NAA and **b** *myo*-inositol concentration in the CNS of WT and AKO mice normalised to the trimethylsilylpropionic internal standard. **c** Overview of onset and rostro-caudal spread of vacuolization and hypomyelination depicted in sagittal sections of the WT and AKO CNS stained with H&E/LFB. **d** High resolution images of H&E and LFB sections of the thalamus demonstrate progressive vacuolization commences after 10 days of age but is readily detectable from P28 onwards.

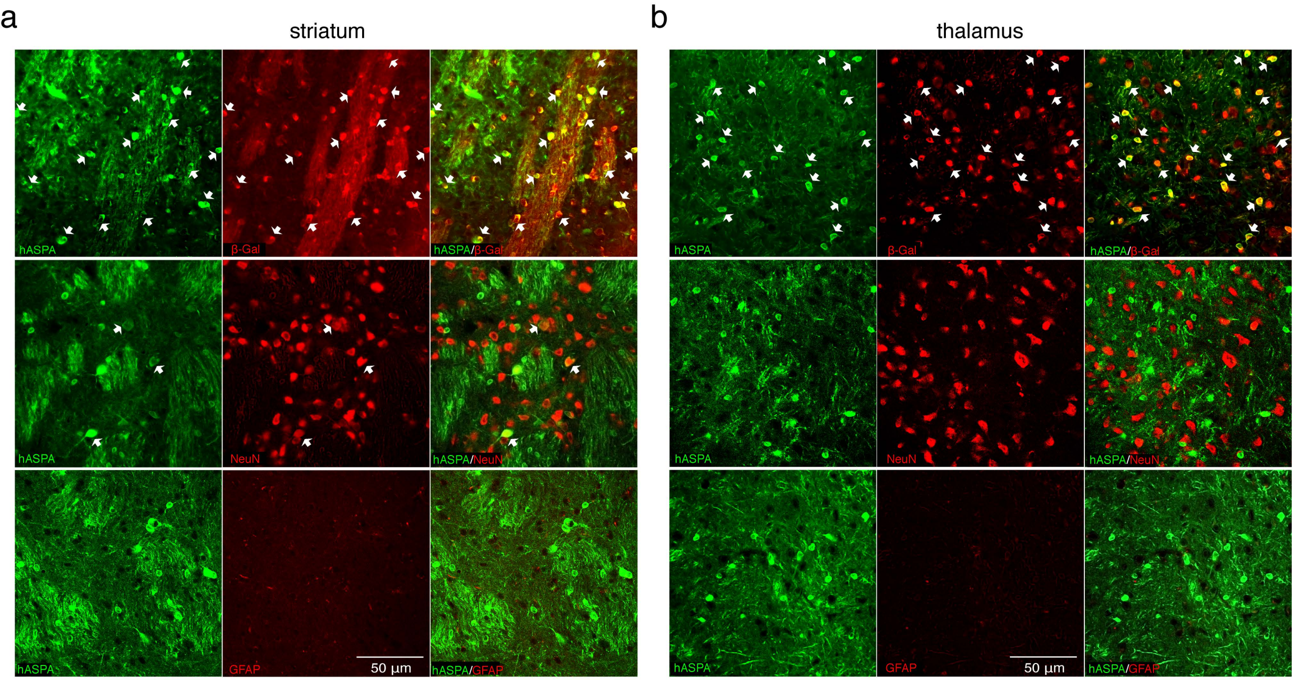
a
striatum
hASPA
β-Gal
hASPA/β-Gal
hASPA
NeuN
hASPA/NeuN
hASPA
GFAP
50 μm
hASPA/GFAP
b
thalamus
hASPA
β-Gal
hASPA/β-Gal
hASPA
NeuN
hASPA/NeuN
hASPA
GFAP
50 μm
hASPA/GFAP

Fig. S8

Immunohistochemistry of coronal sections detected stable expression of transgenic human ASPA eight months following AAV delivery in AKO-GT mice (n = 3). Both in the **a** striatum, a region spared from widespread vacuolisation and the **b** thalamus a severely affected brain region in CD, hASPA (green) was predominantly expressed in oligodendrocytes (top). Targeted disruption of the *Aspa* locus through insertion of the lacZ gene allowed the use β-Gal (red) as reliable oligodendrocyte marker in AKO mice [54]. In contrast limited co-localisation of ASPA with the neuronal marker NeuN (red, middle) was observed and ASPA expression was virtually absent in GFAP positive astrocytes (red, bottom)

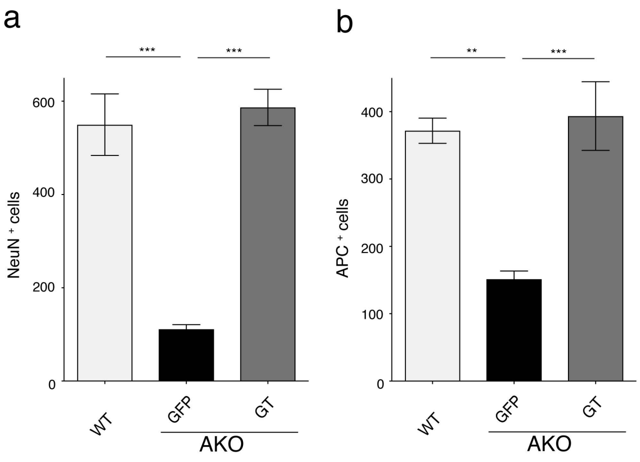
a
b
***
***
**
***
NeuN + cells
APC + cells
0
200
400
600
0
100
200
300
400
WT
GFP
GT
AKO

Fig. S9

AAV-ASPA gene therapy rescues oligodendroglial and neuronal cell loss in the thalamus of AKO mice. Coronal sections of WT, AKO-GFP and AKO-GT treated animals were immunoperoxidase stained for NeuN (neurons) or APC (oligodendrocytes). Cells in 300 μm^2 thalamic sections were counted following DAB mediated detection. The graphed results demonstrate that the number of **a** neurons and **b** oligodendrocytes was restored in the gene therapy cohort ($n = 3$). Data represent mean ± SEM. $^{**}p < 0.01$; $^{***}p < 0.001$; One-way ANOVA with Holm-Sidak *post hoc* test.

	thyNAT	NKO	DKO	*Aspa*$^{\Delta OL}$	AKO
NAA	↑	↓	↓	↑	↑
Juvenile survival[1]	–	–	↓	–	↓
Longevity	–	–	↓	↓	↓
Developmental delay[2]	↑	–	–	–	↑
Adult bodyweight	↓	–	–	↓	↓
Motor behaviour	–	–	–	↓	↓
Neuronal signal propagation[3]	n/a	–	–	n/a	↑
Neuronal recruitment[4]	n/a	↓	↓	n/a	–
Startle response	–	↓	↓	n/a	↓
Sensori-motor gating	–	↓	↓	n/a	–
CNS osmolytes[5]	–	–	–	↑	↑
Vacuolisation	–	–	–	↑	↑
Brain volume	n/a	–	–	–	↑
Myelination	–	–	–	↓	↓
Astrogliosis	–	–	–	↑	↑
Oligodendrocyte #	–	–	–	↓	↓
Neuron #	–	–	–	↓	↓

Table S1: Overview of phenotypic alterations of NAA-mutant mouse strains. Abnormalities compaired with controls are depicted by arrows (no change, black bar; increase, orange; decrease, red). [1]Juvenile survival = number of surviving mice at around postnatal week four; [2]Developmental delay = reduced physical development assessed by attenuated weight gain; [3]Neuronal signal propagation = fVEP and ABR peak latencies; [4]Neuronal recruitment = assessed by growth function of the ABR; [5]CNS osmolytes = organic osmolytes found to be co-regulated with NAA.

Supplementary Experimental Procedures

Ethics statement. All procedures were conducted in accordance with the Australian Code of Practice for the Care and Use of Animals for Scientific Purposes, and were approved by the UNSW Australia Animal Care and Ethics Committee.

Animals. The generation of ASPA-deficient lacZ knock-in *Aspa*$^{lacZ/lacZ}$ mice (denoted AKO) has been described [54]. *Aspa*$^{flox/flox}$ mice were generated via Flp-mediated recombination and deletion of the ß-geo cassette by crossing *Aspa*$^{lacZ/+}$ animals with Flp-deleter mice [63]. *Aspa*^{lacZ} and *Aspa*^{flox} alleles were detected using the same genotyping protocol as described [54]. By utilizing *Cnp*$^{Cre/+}$ mice in which Cre recombinase was expressed from the 2',3'-cyclic nucleotide 3' phosphodiesterase (*Cnp*) gene locus [45], we were able to disrupt the *Aspa*$^{flox/flox}$ allele within cells of the oligodendrocyte lineage (denoted *Aspa*$^{\Delta OL}$). The KO-3582 mutant mouse strain with a targeted deletion of the *Nat8l* gene locus *Nat8l*$^{tm1(KOMP)Vlcg}$ (denoted NKO) was obtained from the Knockout Mouse Project Repository at UC Davis. Genotyping of the *Nat8l* locus was performed by PCR (Neofwd 5′-TCATTCTCAGTATTGTTTTGCC-3′ and SD 5′-CTAGTCCTTCCCTGATGGAG-3′ for the targeted allele, TDF 5′-TGCCATGCTGCACAACTAC-3′ and TDR 5′-ACTCGCGCCCATGTGTCTG-3′ for the wildtype allele). *Aspa*$^{lacZ/lacZ}$; *Nat8l*$^{-/-}$ double-knockout mutants lacking both *ASPA* and *Nat8l* (denoted DKO) were obtained by appropriate crosses between *Aspa*$^{lacZ/+}$ and *Nat8l*$^{-/+}$ mice. In order to restrict the overexpression of *Nat8l* to CNS neurons in transgenic mice (denoted ThyNAT) we blunt-cloned the flag epitope-tagged murine *Nat8l* cDNA [7] into the XhoI site of the

Thy-1.2 minigene cassette [12]. The resulting pThy-Flag-*Nat8l* was linearized by PvuI/NotI, a 7.8kb fragment dissolved at 3 ng / µl in 8 mM Tris-HCl (pH 7.5) / 0.15 mM EDTA, and then microinjected into C57Bl6 x B6D2F1 zygotes as described [36]. Offspring used in this study were backcrossed into C57Bl6 for at least three generations. Hemizygous animals were identified by PCR analysis of genomic DNA isolated from tail biopsies (*Nat8l*_Fwd2: 5′-ATCTTCTACGACGGCATCTTGG-3′ and *Nat8l*_Rev2: 5′-CCCAGTGACTCATAGAGCTTGT-3′). Animals were housed in a temperature-controlled room (21-22°C; 49-55% humidity) with 12 h light-dark cycle (lights on 7:00-19:00). Food and water was available ad libitum.

AAV vector production and stereotaxic delivery

A cDNA encoding human ASPA was subcloned into an AAV2 ITR containing plasmid between the 1.9-kb mouse myelin basic protein (*Mbp*) promoter and the woodchuck posttranscriptional regulatory element (WPRE) followed by a bovine growth hormone poly(A). Packaging and purification of AAV vectors, carrying capsid proteins VP1, VP2 and VP3 from serotype cy5 was performed in human embryonic kidney 293 (HEK) cells as described previously [80]. Genomic titers were determined using the ABI StepOnePlus Real-Time PCR system (Applied Biosystems, Foster City, CA) with primers designed to WPRE [19]. P30 mice were anaesthetised with isoflurane (4% induction, then 1% maintenance with O_2) and placed into a stereotaxic frame (Kopf instruments). Bilateral injection of 1µl containing 2×10^9 vg into each the striatum (+0.7 mm AP, ±1.2 mm ML, −2.6 mm), thalamus (−1.5 mm AP, ±1.0 mm ML, −2.9 mm DV) and cerebellum (−5.0 mm AP, ±1.2 mm ML, −3.1 mm), was performed at 150 nl/min using a microprocessor-controlled mini-pump (World Precision Instruments) with 34G beveled needles (World Precision

Instruments). The needle was left in place for five minutes following injection prior to slow retraction from the brain.

Behavioural testing. Mutants and age- and sex-matched wildtype littermates were used for this study. For open field testing animals were placed in the centre of an arena (40 x 40 x 40 cm^3) under bright light conditions (100 lux) and monitored for 30 min. Total distance travelled and time spent in the centre area and in the periphery was analyzed using the ANY-maze software (Stoetling, Illinois, USA). Briefly, open field boxes were used for recording the locomotor sensitization to cocaine (30 min sessions per test day). After an initial day with saline injection (baseline activity), animals received cocaine (10 mg / kg, IP) for 5 consecutive days (Day 1 - 5; with Day 5 representing the development of sensitization). After 7 consecutive drug- and test-free days, all mice received a priming injection of cocaine (Day 12; representing the expression of sensitization). After another drug-free week (Day 19), locomotion in the absence of the drug was tested again to monitor contextual conditioning.

The rotarod test was performed as described [54]. Briefly, mice were familiarized with the apparatus (Ugo Basile, Comerio, Italy) for four min at constant speed (4 rpm). In a series of 3 trials per animal, the time the mice remained on the accelerating roller (4 - 40 rpm in 4 min) was scored. Between trials the animals were allowed a 30 min period in their home cage. The individual performances were averaged over six trials performed on two consecutive days. For the wire suspension test mice were suspended from a 2 mm wire placed 60 cm above the ground padded with soft embedding material, and the latency to fall was determined. Cut-off time was 60 s. One test-trial was performed to habituate the mice to the apparatus and after a 5 min resting period in their home cage the test was performed. The dowel test was

performed as previously described [33]. Mice were placed onto the middle of a wooden dowel (1 cm diameter), which was placed horizontally about 40 cm above the ground padded with soft embedding material. The latency to fall off the dowel was recorded. The performance was averaged over a series of 3 trials (cut-off time: 2 min) per animal with a 5 min resting period in the home cage between the trials.

Prepulse inhibition (PPI) of the acoustic startle reflex (ASR). Startle testing occurred in a startle chamber (SR-LAB; San Diego Instruments, San Diego, USA) as described previously [65]. PPI was calculated as the per cent decrease of the ASR magnitude in trials when the startle stimulus was preceded by a prepulse [100 x (mean ASR amplitude on pulse alone trials – mean ASR amplitude on prepulse-pulse trials)/mean ASR amplitude on pulse alone trials].

Cochlear response recordings. Auditory function was assessed by determining auditory brainstem response (ABR) in a sound attenuating. Animals of both sexes at 10 weeks were used. The ABR threshold following click stimulus was determined as the intensity at which an ABR P2 wave could still just be visually detected above the noise floor. ABR input-output functions were determined after chamber as described previously [79].

Visual evoked potentials. Flash and pattern reversal visual evoked potentials (fVEP) were recorded from animals at three months as described [70]. Briefly, mice were anaesthetised using a ketamine/xylazine/acepromazine (40 mg / kg, 8 mg / kg, 0.5 mg / kg; i.p.) and eye ointment was applied to each eye to prevent dryness. Binocular flash stimulus (white light, 1 / sec, 10 us duration, 250 ms sampling) presented from a

digital xenon stroboscope (Industrial Equipment and Control Pty. Ltd, Thornbury, VIC, Australia) 10 cm in front of the nose of the mouse was filtered with a low pass filter of 3 kHz. Recordings (minimum of 300 stimuli averages) were collected using subdermal platinum electrodes (midline, just distal to the interorbital line, negative electrode; midline, nuchal crest, positive electrode; near hind leg, ground electrode) in a darkened sound-attenuating chamber (Sonora Technology, Japan) using the TDT system 3 with RX6 and RX6-2 signal processors (Tucker David Technology, Ft Lauderdale, FL, USA) and BioSig32 software.

Whole body composition analysis. Body composition measurements were performed in conscious animals using the EchoMRI-900™ with A100 mouse antenna insert (Echo Medical Systems, Houston, TX USA).

Magnetic resonance imaging. Prior to all in vivo MR imaging procedures, general anaesthesia was induced in an induction chamber with 4 % isoflurane in oxygen (1 L / min). Animals were then transferred to the scanner animal bed and received 1 - 2.5 % isoflurane at 0.8 l / min oxygen flow rate through a nose cone to maintain anaesthesia during the scanning procedure. Respiratory motion was monitored during the imaging procedures using a pressure sensitive pad. Animal body temperature was maintained at 37 °C by a temperature controlled circulating water warming blanket. *In vivo* imaging was performed on a 9.4 T Bruker (Ettlingen, Germany) BioSpec Avance III 94/20 magnetic resonance microimaging system equipped with BGA-12S HP gradients with maximum strength 660 mT / m and slew rate 4570 Tm / s. A dedicated 23 mm internal diameter quadrature brain volume coil was used for radiofrequency transmission and reception.

Images were acquired with an optimized 2D T2w brain anatomy protocol using Rapid Acquisition with Relaxation Enhancement (TurboRARE) with 8 echos per echo-train. Imaging slices covered the complete brain region in axial orientation with the following parameters: TE_{eff} = 43 ms, TR = 2500 ms, FOV = 15 × 15 mm, acquisition matrix = 256 × 128 mm, in plane resolution = 59 × 118 µm, slice thickness = 0.5 mm, slice gap = 0.5 mm, 18 axial slices, 10 averages, resulting in a total scan time of 620 s. For a more detailed volumetric analysis a single animal of each group also received an additional high resolution scan with an isotropic 3D T2w TurboRARE protocol with the following parameters: 8 echoes per echo-train, TE_{eff} = 33 ms, TR = 1800 ms, FOV = 20 x 15 × 15 mm, acquisition matrix = 200 × 150 x 150 mm, isotropic resolution = 100 x 100 x 100 μm^3, overall acquisition time 34 min (untriggered). To minimize artefacts all 3D acquisitions were triggered on the respiratory signal from the animal monitoring system. For volumetric and statistical analysis all images were exported from the scanner as 2D Dicom slices and subsequently converted into image volumes in NIfTI file format. Whole brain and ventricle structures were segmented using thresholding and delineation methods provided by the 3D slicer package [22]. Surface models were generated from each segmentation to determine volume and surface for the respective structures. *In vivo* ^{1}H-MR spectroscopy procedures was performed under general anaesthesia as above on a 9.4 T Bruker BioSpec Avance III 94/20 magnetic resonance microimaging system (Bruker) equipped with a cryogenic very low temperature, closed cycle cooled RF-coil. Spectra from a 2×2×3 mm^3 voxel in the thalamus were acquired at an echo time of 10 ms using a PRESS single voxel sequence as described in [54]. Automatic quantification of the spectra was performed using the LC model (version 6.3).

Immunofluorescence detection of antigens in brain sections. Briefly, mice were deeply anesthetized with pentobarbital and trans-cardially perfused with phosphate buffered saline (PBS), followed by 10% Neutral buffered formalin (NBF; Sigma). Brains were removed and post-fixed in NBF (2 h), followed by cryo-protection in 30 % sucrose / PBS and cut into free-floating sections at 40 μm using a cryostat. Sections were stored at 4 °C in cryoprotection solution (25 % glycerin, 25 % ethylene glycol and 50 % PBS) until use. If required, antigen retrieval preceded permeabilization, by rinsing free floating sections twice in PBS followed by incubation in 10 mM sodium citrate buffer (pH 6.0) containing 0.1 % Tween 20 at 80 °C for 30 min. Sections were allowed to cool down to room temperature in the same solution followed by permeabilization with 0.1 % TritonX-100 in PBS (PBS-Tx), and block of non-specific binding with 4 % normal horse serum (NHS) in PBS-Tx. Sections were incubated overnight at 4 °C with a combination of the following antibodies in 4 % NHS in PBS-Tx: rabbit anti-ASPA serum (1:1000, developed in-house [54]), mouse anti-NeuN (1:500; Millipore #mab377), rat anti-MBP (1:1000; abcam #ab62631), mouse anti-GFAP (1:5000; Cell signalling #3670), rat anti-MBP (1:1000; Abcam no. ab7349), chicken anti-β-Gal (1:1000: Abcam #9361), mouse anti-Flag (1:200; Cell Signalling #2146), rabbit anti-Neurofilament 200 (1:4000; Sigma # N4142). Sections were then washed with PBS and incubated with appropriate Alexa-488/594 conjugated secondary antibodies (1:1000, Thermo Fisher) for 4 h at room temperature in 4% NHS in PBS-Tx. Following two washes in PBS for 10 min, sections were incubated in the nuclear dye 4',6-diamidino-2-phenylindole (DAPI). After another wash in PBS-Tx, sections were mounted with Mowiol (Calbiochem). Fluorescence was visualized using a Zeiss Z1 AxioExaminer NLO710 confocal microscope (Carl Zeiss MicroImaging). Quantitative analysis of transgenic ASPA expressing cells was

performed using double-immunofluorescence with lineage specific markers as described in [78]. β-Gal has previously been shown to be a reliable marker for oligodendrocytes in AKO mice [54] and NeuN for neurons and GFAP for astrocytes. The percentage of human ASPA expressing cells in the striatum and thalamus was determined by counting at least at least 150 hASPA positive cells from a minimum of three non-adjacent sections in a minimum of three biological replicates.

Immunoperoxidase detection of antigens in brain sections and lineage positive cell quantification. Briefly, comparable 40 µm free floating sections located between -1.4mm and -1.8mm from Bregma containing the thalamus were washed in PBS. For APC immunohistochemistry, sections were subjected to a 30 min heat - induced antigen retrieval in 10mM sodium citrate supplemented with 0.05% TWEEN 20 (pH 6.0) at 95 °C. Following gradual cooling and three consecutive washes in PBS section were incubated in 50% Methanol supplemented with 3 % H_2O_2 for 30 min. After repeated washes in PBS sections were blocked in blocking buffer containing 2% bovine serum albumin (Sigma), 3% normal horse serum (HS) and 0.1 % TritonX-100 in PBS for 3 hours. Primary antibody incubation was performed at 4 °C using either rabbit anti-NeuN (Cell Signalling #12943) diluted 1:500 or mouse anti-APC (Merck OP80) diluted 1:20 in 0.5% PB with 2% HS. Following three washes in PBS the appropriate biotinylated secondary antibody (Dianova) was applied in a 1:1000 dilution in 0.5% PB with 2% HS. Diamminobezidine (DAB) detection was performed using a Vectastain Elite ABC kit (Vector Labs) according to the manufacturers instructions [15]. Quantification of lineage positive cells was performed in at least two 300 μm^2 sections of comparable regions in the thalamus slices using the 'cell counter' plugin for Image J.

Haematoxylin & Eosin (H & E) staining and Luxol Fast Blue (LFB) staining. Animals were killed by CO_2 or transcardiac perfusion. Following decapitation the brains were isolated and immediately fixed in 10 % Neutral Buffered Formalin (NBF; Sigma) for 48 hrs. Brains were processed on a Sakura VIP6 Auto processor followed by embedding in paraffin using a Sakura Tissue-Tek Embedder. Tissues were sectioned at 5 µm (Zeiss Hydrax M40) and stained on an Leica Auto Stainer CV5030 using Harris haematoxylin and 1 % alcoholic Eosin. LFB staining was performed on brain sections using the Kluver and Barrera method. Briefly, paraffin sections were dewaxed followed by treatment in 100% ethanol. Then sections were incubated overnight in LFB solution (0.1 % LFB in 95 % ethanol / 0.5 % acetic acid) at 60 °C. Following washes in 95 % ethanol and ddH_2O the staining was differentiated in 4 % lithium carbonate for 2 - 4 sec. Differentiation commenced in 70 % ethanol until the grey matter was colourless and white matter appeared blue. Sections were then rinsed in ddH_2O before counterstaining with either preheated 0.025 % Cresyl Violet acetate solution for 10 min at 37 °C, or with H & E. Finally, sections were rinsed in ddH_2O, dehydrated, cleared and coverslipped. Stained sections were digitized using Mirax (Carl Zeiss) or Aperio (Leica) slide scanners.

Immunoblotting. Animals were killed at 6 - 9 months, organs dissected quickly and snap frozen. Brains were homogenized under liquid nitrogen using mortar and pestle. Aliquots were sonicated in solubilization buffer (30 mM Tris-HCl, pH 7.4, and 5 mM $MgCl_2$ containing 4 mg of CHAPS (Sigma) and 20 % glycerol) containing protease inhibitors (Complete, Roche Applied Science) and protein concentration was determined by the method of Bradford. 20 µg of protein were mixed with 5x Laemmli

reducing sample buffer, denatured for 5 min at 95 °C (with exception of samples allocated for detection of PLP), separated by SDS-PAGE and transferred onto PVDF membranes (Protran, Whatman; GE Healthcare). Membranes were probed with the following antibodies: rabbit anti-ASPA serum (1:1000), mouse anti-GAPDH (1:5000, Sigma, G8795), rat anti-MBP (1:1000; Abcam, ab7349), rat anti-PLP aa3 (1:200, gift of J. Trotter), rat anti-NG2 (1:100, J. Trotter). Antibodies were detected by the appropriate HRP-conjugated secondary antibodies (Dianova) followed by using the enhanced chemiluminescence system (BioRad) and digitalized.

^{1}H Nuclear magnetic resonance (NMR) spectroscopy. Animals were killed by cervical dislocation, followed by quick dissection of the cortex, cerebellum, brainstem, kidney and liver, immediately snap freezing dissected tissue in liquid nitrogen. Brain tissues were pulverized, solvent extracted [46] and the aqueous phase lyophilized. The pellet was retained to determine protein concentration. The lyophilized extract was stored at -20 °C until required for NMR analysis. The lyophilized extract was reconstituted in 0.17 ml $^{2}H_2O$ containing 2 mM [^{13}C] formate as an internal intensity reference, and 6 mM EDTA to chelate paramagnetic ions and remove localized field inhomogeneity, particularly for the citrate resonance. All spectra were acquired on a Bruker AVANCE III HD 600 spectrometer fitted with a cryoprobe (TCI) and refrigerated sample changer. ^{1}H spectra were acquired, both with and without decoupling ^{13}C using bilev composite pulse decoupling, across an effective bandwidth of 48000 Hz during the acquisition time, on a 30 s duty cycle. Total metabolite pool sizes were determined using TOPSPIN (v3.1) from the $^{1}H\{^{13}C$-decoupled$\}$ spectra as described previously [61].

Myelin lipid quantification. Animals were killed by cervical dislocation and the brain dissected. Briefly, the hindbrain was separated with a scalpel blade and brainstem detached from the cerebellum. The cortex was carefully peeled off from subcortical layers of the remaining brain. Brain regions were snap frozen in liquid nitrogen immediately following dissection. Tissue was pulverized and spiked with d18:1/12:0 galactosylceramide (250 pmol), d18:1/12:0 sulfatide (250 pmol) internal standards. Lipids were extracted using a two phase protocol [46]. Briefly, 1 unit (mass) tissue, was mixed with 2 units (volume) methanol and 1 unit (volume) chloroform. The mixture was left on ice for 15 minutes for the chloroform to penetrate. Half a unit of chloroform and half a unit of ddH_2O was added to separate the aqueous and lipophilic phases. Following centrifugation, the first extraction was performed by removing the upper aqueous. Then, three parts methanol and two parts ddH_2O were added to the lipophilic phase. The tissue was left for 15 min, half a unit of chloroform and half a unit of ddH_2O was added to separate the aqueous and lipophilic phases. Following another centrifugation, the second extraction was performed by removing upper aqueous phase and adding it to the first extraction. Extracts were dried under vacuum and reconstituted in HPLC mobile phase: 80 % methanol / 20 % deionised water containing 0.2 % formic acid and 1 mM ammonium formate, then stored at -20 °C until analysis. Lipids were quantified using a TSQ Access triple quadruple mass spectrometer (Thermo Fisher Scientific) operating in positive ion mode, as described [82]. Peak identity was verified using both column elution time and precursor and product ion mass pairs, as described [35]. Lipids, expressed as ratios to the relevant internal standard, were quantified using standard curves prepared with GalCer, and sulfatide external standards (Avanti Polar Lipids).

Quantification of NAAG levels. NMR Samples were dried and resuspended in 200 uL of 95:5 acetonitrile:water for analysis. LC-MS/MS was performed on using a CTC PAL Autosampler, Accela UHPLC and Quantum Access mass spectrometer (ThermoFisher Scientific). 20 uL of sample was injected onto a Merck-Sequant zic-HILIC column (2.1 x 100 mm) and the analytes separated at a flow rate of 500 uL / min using a gradient of 5mM ammonium acetate, 0.1 % formic acid in MilliQ water (A) against 0.01 % formic acid in acetonitrile (B), (Time 0 = 95 % B, 0.7 = 95 % B, 4 = 5 % B, 4.3 = 5 % B, 4.5 = 95 % B, 10 = 95 % B). Column eluate was directed to the heated electrospray ionisation probe of the mass spectrometer operating in positive mode. Source conditions and Selected Reaction Monitoring (SRM) transitions were optimised prior to analysis using direct infusion of purchased NAAG compounds (Sigma Aldrich). NAAG was quantified using the transition 305 > 130. Additional transitions were included in the method as qualifiers but not used for quantification. External calibration curves were constructed using 7 points between 0 and 100 ng. Samples were quantified directly against this curve with no use of internal standard.

RNA isolation and Q-PCR. Animals were killed, organs dissected quickly, and snap frozen. Brains were homogenized under liquid nitrogen using mortar and pestle. RNA was extracted following the manufacturer's instructions (RNeasy MiniKit, Qiagen Pty Ltd). DNase treatment was performed using on-column DNase digestion and RNA was eluted in 30 μl RNAse free water. Following determination of RNA content, integrity and purity [NanoDrop ND-1000 (NanoDro Technologies, Inc., Wilmington, DE, USA) and agarose gel electrophoresis], cDNA was obtained from 500 ng total RNA according to the manufacturer's instructions (Applied Biosystems Mulgrave,

VIC, Australia). The cDNA equivalent to 22.5 ng RNA was amplified by quantitative reverse transcription PCR utilizing TaqMan assays (Applied Biosystems, Carlsbad, CA) for Aspartoacylase (*Aspa*; Mm00480867_m1), *Nat8l* (Mm01217216_m1), NAAGS-I (*Rimklb*; Mm01184191_m1), NAAGS-II (*Rimkla*; Mm00616927_m1), Hypoxanthine phosphoribosyltransferase (*Hprt*; Mm00446953_m1) using an ABI7700 real time PCR cycler (Applied Biosystems Mulgrave, VIC, Australia). Quantitative RT-PCR reactions were performed in triplicates. The comparative CT method (ΔΔCT) for relative quantification of expression was used. Data obtained were normalized using Hprt as a control transcript. The normalized expression value (ΔCT) for samples obtained from mutants was compared to the equivalent ΔCT for the control samples. The ΔCT-Ctr is calculated as follows: ΔCT-Ctr = (CT-Ctr with Hprt primers in controls) – (CT-Ctr with Hprt primers in mutants). The normalized expression values were compared in mutant samples and controls by calculating ΔΔCT: ΔΔCT = ΔCT mutant - ΔCT control.

Statistics. Generally, graphs and statistical analyses were done with GraphPad Prism 6.0 software. Student's t-test, One-Way or Two-Way ANOVA followed by Tukey post-hoc test was used for statistical analysis as appropriate. Values are presented as the mean ± s.e.m and $p < 0.05$ was considered as statistically significant. For multivariate analysis. values of total lactate, aspartate, glutamine, creatine, taurine and *myo*-inositol, glutamate and GABA derived from ^{1}H NMR spectra of WT, AKO, NKO and DKO mice were imported into Simca-P+ software package (v11.5, Umetrics, Umeå, Sweden). Data were univariance scaled to standardize variance between the high and low concentration metabolites [83], ensuring that variables contributed equally to the model. These data formed a 3 component model accounting

for 98% of the variance in the data (71, 23 and 4 % for principal components 1,2 and 3, respectively), with a cross validation score (Q2) of 0.90.

Declaration. The authors confirm that all data are available on request.
